# Trajectory tracking control of unmanned aerial vehicle with disturbance observer and robust adaptive neural dynamic surface

**DOI:** 10.1371/journal.pone.0358433

**Published:** 2026-09-18

**Authors:** Xian Pan, Dongxue Wang, Youwu Liu, Xiang Nan

**Affiliations:** 1 Guangzhou College of Technology and Business, Guangzhou, China; 2 School of Information Engineering, Henan Mechanical and Electrical Vocational College, Zhengzhou, China; 3 School of Economics and Management, Sanming University, Sanming, China; 4 Nantong Normal College, Nantong, China; Guangdong University of Technology, CHINA

## Abstract

This paper investigates the trajectory tracking control problem for a class of uncertain strict feedback nonlinear systems subject to unknown dynamics and time varying external disturbances, with a specific application to unmanned aerial vehicle (UAV) longitudinal motion. A novel robust adaptive neural dynamic surface control (DSC) scheme integrated with nonlinear disturbance observers (DOB) is proposed. First, nonlinear disturbance observers are systematically constructed for each subsystem to provide real-time estimates of unknown bounded disturbances, and these estimates are explicitly incorporated into both virtual and actual control laws for active compensation, significantly enhancing disturbance rejection capability. Second, radial basis function (RBF) neural networks are employed to approximate the unknown continuous functions arising from system dynamics, and a parameter aggregation strategy is adopted to reduce the number of online adaptation parameters, thereby simplifying the implementation. Third, the dynamic surface control technique is utilized to overcome the “explosion of complexity” inherent in conventional backstepping designs, eliminating the need for analytical differentiation of virtual control laws. Numerical simulations demonstrate superior tracking accuracy and disturbance rejection capability compared to conventional adaptive neural DSC without disturbance observers, validating the effectiveness and robustness of the proposed scheme.

## 1 Introduction

In the past decade, the application of unmanned aerial vehicle (UAV) in civilian and commercial fields has seen unprecedented growth, from precision agriculture and environmental monitoring to search and rescue operations [1–3]. Due to the increasing complexity of mission profile, more and more strict requirements are put forward for flight control system [4], especially in the aspects of trajectory tracking accuracy, robustness against environmental interference and adaptation to flight conditions [5]. However, the control of UAV is still a well-known problem because of their inherent characteristics: strong cross-coupling between longitudinal and lateral modes [6], significant parameter uncertainty caused by highly nonlinear aerodynamic changes [7], and susceptibility to external disturbances such as gust and atmospheric turbulence [8]. The fact that the precise mathematical model of UAV dynamics is usually unavailable or too complicated for real-time control implementation further aggravates these challenges, and it is necessary to develop control strategies that can operate effectively under significant model uncertainty.

The longitudinal dynamics of fixed-wing UAV constitutes a typical nonlinear system with strict feedback [9], and its trajectory tracking control becomes an ideal platform to test the nonlinear control theory because of the existence of unknown aerodynamic parameters and external disturbances [10]. In the past decades, people have studied various nonlinear control technologies to realize trajectory tracking and attitude adjustment of UAV [11–13]. In [14], Zhao et al. proposed a nonlinear robust adaptive control strategy based on the immersion and invariance methodology, which achieves asymptotic tracking without requiring precise convergence of parameter estimates. Based on fuzzy adaptation and neural compensation, Mendoza and Yu developed a trajectory tracking control law for multi-rotor UAVs, in which PID parameters are tuned online by fuzzy rules to effectively handle model uncertainties and external disturbances [15]. However, these methods rely heavily on the accurate elimination of nonlinear terms, so they show poor robustness when there are model uncertainties. Sliding mode control (SMC) is inherently robust to matching uncertainty and interference. Because of its simplicity and strong interference suppression ability, SMC has been widely used in UAV control [16–18]. To address the trajectory tracking control problem of quadrotor UAVs under uncertain environment parameters, Hou et al. [19] proposed an adaptive robust controller based on the backstepping sliding mode method, which achieves improved tracking accuracy and robustness against disturbances. In [17], Baek and Kang proposed a synthesized SMC method that effectively suppresses chattering while achieving high-precision attitude tracking performance. However, the discontinuous switching characteristics of SMC will inevitably introduce chattering, which may excite unmodeled high-frequency modes and lead to excessive wear of actuators. In recent years, the integration of adaptive neural networks with dynamic surface control has emerged as a promising direction for UAV systems under complex operational conditions. Zhu and An [20] investigated adaptive neural network formation secure control for quadrotor UAVs under wind disturbances and denial-of-service attacks, demonstrating the effectiveness of neural approximation in handling both environmental uncertainties and cyber threats. For nonlinear systems with unknown dynamics, Li et al. [21] developed an event-triggered optimal control framework combining disturbance observer-based estimation with reinforcement learning backstepping, achieving significant communication savings while maintaining control performance. Gu et al. [22] proposed a finite-time prescribed performance tracking control scheme based on a novel neural network disturbance observer, ensuring both rapid convergence and guaranteed transient performance for strict-feedback nonlinear systems.

In various nonlinear control paradigms, inversion has become one of the most systematic and powerful methods for strict feedback systems, providing a recursive design process with built-in Lyapunov stability certificate [23]. Backstepping has been successfully applied to many UAV control problems, including attitude tracking [24], formation flying [25] and path tracking [26]. In [27], Chen et al. proposed a nonlinear resilient control method integrating backstepping control with a nonlinear disturbance observer, which effectively attenuates disturbances and ensures system stability and tracking performance. To solve the trajectory tracking control problem of quadrotor UAV under the influence of random disturbance, Yu et al. [28] proposed a finite-time adaptive fuzzy backstepping control method, which can ensure the system to achieve finite-time convergence in the random disturbance environment and significantly improve the rapidity and robustness of tracking response. Based on the constraint handling mechanism of obstacle Lyapunov function and the recursive design of adaptive backstepping control, Khadhraoui and others developed a robust adaptive control scheme for quadrotor UAV, which can estimate the unknown parameters of the system online and actively manage the state constraints, and maintain reliable tracking performance under complex flight conditions [29]. However, one of the main shortcomings of traditional backstepping is the well-known “complexity explosion” problem: the virtual control law must be analytically differentiated at each step, which leads to the rapid increase of the complexity of the expression with the increase of the system order [30]. To circumvent the explosion of complexity, Swaroop et al. [31] introduced the dynamic surface control (DSC) technique, which replaces the analytical differentiation of virtual controls with first-order low-pass filters. Recent extensions of DSC have addressed increasingly complex scenarios, including predefined-time cooperative formation control for tandem-rotor UAVs [32] and filter-based intelligent output-constrained control for uncertain MIMO systems with sensor and actuator faults [33]. Moreover, the integration of disturbance observers with neural adaptive DSC has been explored for UAV formation control under cyber-attacks [34] and for tiltrotor UAV attitude control with actuator faults [35], demonstrating the effectiveness of combining disturbance observation with DSC for enhanced robustness. In [36], Huang et al. proposed an adaptive dynamic surface control method based on a hybrid event-triggering mechanism, which significantly reduces controller update frequency and communication resource consumption while ensuring system stability and tracking accuracy. To improve the heading control security of USVs in environments with limited communication resources and potential cyber attacks, Zhao et al. [37] designed a dynamic event triggered resilient control framework that employs encrypted data transmission and adaptive triggering thresholds to reduce unnecessary communications while actively defending against cyber threats. The work of Shao et al. [38]addresses the disturbance rejection problem in quadrotor UAV trajectory tracking, proposing a dynamic surface control method based on an extended state observer that treats external disturbances, unmodeled dynamics, and parameter uncertainties as a unified lumped disturbance for estimation and compensation, significantly enhancing the robustness of the control system. More recently, Ma et al. [39] developed a hyperchaotic privacy-preserving cooperative adaptive control framework for multimotor systems, where hyperchaotic signals are exploited to encrypt system states during network transmission, achieving both synchronization control and data security in a unified architecture. Peng et al. [40] investigated the edge-based dynamic event-triggered inverse optimal formation control problem for multiple quadrotor UAVs with attitude constraints, establishing a distributed control framework that simultaneously optimizes communication efficiency and formation performance through inverse optimality. These works showcase the growing trend of integrating advanced control objectives—such as privacy preservation, communication reduction, and formation coordination—with adaptive control methodologies for complex multi-agent systems.

Despite the aforementioned advances, fixed-wing UAV trajectory tracking under practical conditions presents three fundamental challenges that existing methods fail to address simultaneously: (i) aerodynamic coefficients are uncertain and flight-condition-dependent, precluding accurate model based design; (ii) external disturbances are time varying and persistent, rendering neural approximation alone insufficient for effective rejection; (iii) the strict-feedback structure demands recursive design, where repeated differentiation of virtual controls causes rapid complexity growth. Most existing adaptive neural schemes assume matched disturbances, require known control gain bounds, or overlook the coupling between approximation errors, disturbance residuals, and filtering errors in stability analysis. A systematic framework combining disturbance observers with neural DSC for fixed-wing UAVs under concurrent unknown dynamics, time-varying disturbances, and uncertain control gains has yet to be rigorously established.

The engineering application of DSC in UAV system is limited by the dependence of virtual control law on the nonlinear prior knowledge of the system, and RBF neural network has become the mainstream technical means to compensate this limitation because of its universal approximation ability and simple parameter linear structure [41]. Motivated by the above observations, this paper proposes a disturbance-observer-based adaptive neural dynamic surface control scheme for a class of uncertain strict-feedback nonlinear systems, with a specific application to UAV longitudinal trajectory tracking. The main contributions of this work are fourfold:

1. Nonlinear disturbance observers are constructed for each subsystem to estimate unknown time-varying disturbances, with the estimates explicitly embedded in both virtual and actual control laws for active compensation, thereby enhancing robustness beyond neural approximation alone.
2. RBF neural networks approximate unknown smooth system functions, while a lumped parameter aggregation strategy reduces the number of online adaptation parameters and simplifies implementation. The σ-modification term in the adaptive laws ensures boundedness of weight estimates.
3. A Lyapunov-based stability analysis is developed to establish semi-global uniform ultimate boundedness (SGUUB) of all closed-loop signals, explicitly accounting for the coupling among neural approximation errors, disturbance estimation residuals, and dynamic surface filtering errors. Clear tuning guidelines for design parameters are provided.
4. The proposed framework is validated through application to fixed-wing UAV longitudinal trajectory tracking with unknown aerodynamic coefficients and external disturbances. Comparative simulations against conventional adaptive neural DSC without disturbance observers demonstrate superior tracking accuracy and disturbance rejection.

The remainder of this paper is organized as follows. Section II presents the problem formulation, including the system description, the UAV longitudinal model, necessary assumptions, and preliminaries on RBF neural networks. Section III details the design of nonlinear disturbance observers and the adaptive neural DSC law with disturbance compensation. Section IV provides the rigorous stability analysis. Section V presents simulation results and discussions. Section VI concludes the paper and outlines future research directions.

## 2 UAV longitudinal dynamics

The longitudinal motion of a fixed-wing UAV in the pitch plane is governed by the following set of differential equations, which describe the evolution of the flight path angle, angle of attack, pitch angle, and pitch rate [42]:


$$\begin{array}{c} \dot{\gamma }={\bar{L}}_{\alpha }\alpha -\frac{g}{V_{T}}\cos\gamma +{\bar{L}}_{0}, \end{array}$$ (1)

$$\begin{array}{c} \dot{\alpha }=q+\frac{g}{V_{T}}\cos\gamma -{\bar{L}}_{0}-{\bar{L}}_{\alpha }\alpha , \end{array}$$ (2)

$$\begin{array}{c} {\dot{\theta }}_{p}=q, \end{array}$$ (3)

$$\begin{array}{c} \dot{q}=M_{0}+M_{q}q+M_{\delta }\delta , \end{array}$$ (4)

where γ is the flight path angle, α the angle of attack, $\theta_{p}$ the pitch angle, $q={\dot{\theta }}_{p}$ the pitch rate, δ the elevator deflection serving as the control input, $V_{T}$ the true airspeed maintained constant by an inner-loop controller, *g* the gravitational acceleration, and ${\bar{L}}_{0},{\bar{L}}_{\alpha },M_{0},M_{q},M_{\delta }$ the aerodynamic lift and pitching moment coefficients.

**Fig 1:**
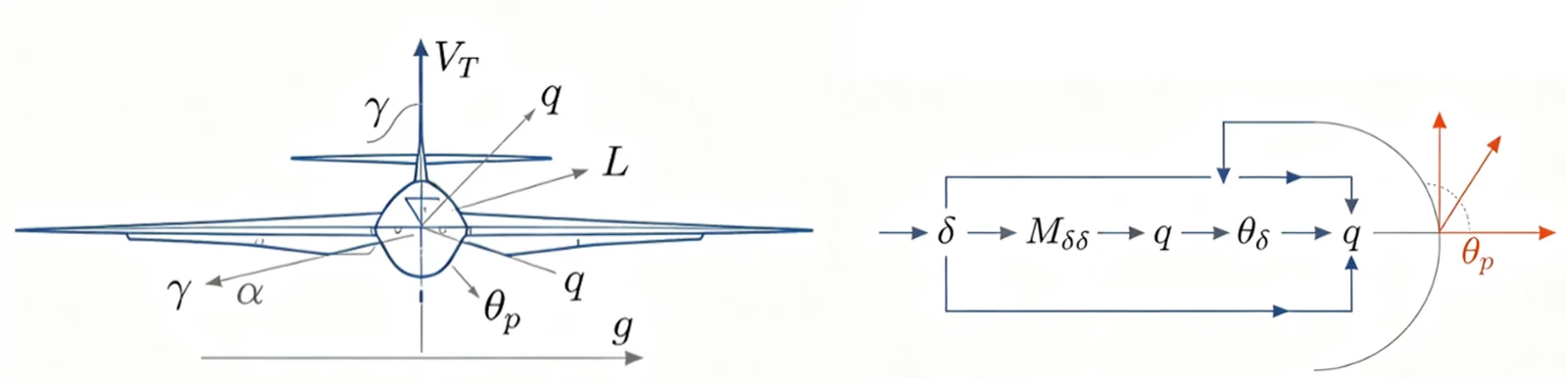
Geometric illustration of the UAV longitudinal motion.

**Remark 1:** The relationship among the three angles is $\theta_{p}=\gamma +\alpha$ for longitudinal flight, as shown in Fig 1. However, in the simplified model considered here, the pitch angle equation is retained separately to illustrate the kinematic relation. For control design purposes, the state variables are selected as (γ,α,q), which form a complete representation of the longitudinal dynamics.

The aerodynamic coefficients ${\bar{L}}_{0}$, ${\bar{L}}_{\alpha }$, *M*_0_, $M_{q}$, and $M_{\delta }$ are generally unknown functions of the flight condition. In this study, at a given operating point, these coefficients are treated as unknown constants. This assumption is reasonable for small perturbations about a trim condition, which is common in many UAV flight control scenarios. The lift-related terms are:


$$\begin{array}{c} {\bar{L}}_{0}=\frac{L_{0}}{mV_{T}},\;{\bar{L}}_{\alpha }=\frac{L_{\alpha }}{mV_{T}}, \end{array}$$ (5)

where *L*_0_ represents the lift component independent of angle of attack, $L_{\alpha }$ is the lift curve slope, and *m* is the UAV mass.

The pitching moment *M*_0_ in (4) includes contributions from the aerodynamic center and static stability, while $M_{q}$ accounts for pitch damping effects. The term $M_{\delta }\delta$ represents the control moment generated by elevator deflection. Typically, $M_{\delta }>0$ for positive elevator effectiveness.

**Remark 2:** The airspeed $V_{T}$ is assumed to be regulated by a lower-level controller and thus treated as constant. This is a standard assumption in the literature for longitudinal trajectory tracking, as the speed dynamics evolve on a faster timescale compared to the attitude dynamics. Under this assumption, the longitudinal and lateral dynamics become decoupled, allowing independent control design.

### 2.1 Transformation to strict-feedback form

To design a systematic control law, we transform the UAV longitudinal model (1)–(4) into a more amenable strict-feedback representation. Define the state variables:


$$\begin{array}{c} x_{1}=\gamma ,\;x_{2}=\alpha ,\;x_{3}=q, \end{array}$$ (6)

and the control input:


$$\begin{array}{c} u=\delta . \end{array}$$ (7)

Then, the dynamics can be rewritten as:


$$\begin{array}{c} {\dot{x}}_{1}={\bar{L}}_{\alpha }x_{2}-\frac{g}{V_{T}}\cos x_{1}+{\bar{L}}_{0}, \end{array}$$ (8)

$$\begin{array}{c} {\dot{x}}_{2}=x_{3}+\frac{g}{V_{T}}\cos x_{1}-{\bar{L}}_{0}-{\bar{L}}_{\alpha }x_{2}, \end{array}$$ (9)

$$\begin{array}{c} {\dot{x}}_{3}=M_{0}x_{2}+M_{q}x_{3}+M_{\delta }u. \end{array}$$ (10)

Comparing (8)–(10) with the standard strict-feedback form, we can identify the following unknown functions and parameters:


$$\begin{array}{c} g_{1}={\bar{L}}_{\alpha }>0, \end{array}$$ (11)

$$\begin{array}{c} f_{1}(x_{1})=-\frac{g}{V_{T}}\cos x_{1}+{\bar{L}}_{0}, \end{array}$$ (12)

$$\begin{array}{c} f_{2}(x_{1},x_{2})=\frac{g}{V_{T}}\cos x_{1}-{\bar{L}}_{0}-{\bar{L}}_{\alpha }x_{2}, \end{array}$$ (13)

$$\begin{array}{c} f_{3}(x_{2},x_{3})=M_{0}x_{2}+M_{q}x_{3}, \end{array}$$ (14)

$$\begin{array}{c} g_{3}=M_{\delta }>0. \end{array}$$ (15)

Substituting the definitions above, the UAV dynamics take the following strict-feedback form:


$$\begin{array}{c} {\dot{x}}_{1}=g_{1}x_{2}+f_{1}(x_{1}), \end{array}$$ (16)

$$\begin{array}{c} {\dot{x}}_{2}=x_{3}+f_{2}(x_{1},x_{2}), \end{array}$$ (17)

$$\begin{array}{c} {\dot{x}}_{3}=f_{3}(x_{2},x_{3})+g_{3}u. \end{array}$$ (18)

**Remark 3:** In the nominal model (16)–(18), the strict-feedback structure is clearly visible: each state equation depends only on the current state and the next state, which serves as a virtual control input. The control input *u* appears only in the last equation, and the system has relative degree three. This triangular structure is precisely the class of systems for which backstepping and dynamic surface control are designed.

### 2.2 Incorporation of uncertainties and disturbances

In practice, the UAV dynamics are subject to various sources of uncertainty, including:

- Parametric uncertainties in the aerodynamic coefficients ${\bar{L}}_{0},{\bar{L}}_{\alpha },M_{0},M_{q},M_{\delta }$;
- Unmodeled dynamics such as structural flexibility, actuator dynamics, and sensor noise;
- External disturbances such as wind gusts, atmospheric turbulence, and ground effect.

To account for these factors, we augment the nominal model (16)–(18) with additive disturbance terms $d_{i}(t)$ and consider the unknown functions $f_{i}$ as entirely unknown, rather than their explicit forms in (12)–(14). The uncertain system is thus written as:


$$\begin{array}{c} {\dot{x}}_{1}=g_{1}x_{2}+f_{1}(x_{1})+d_{1}(t), \end{array}$$ (19)

$$\begin{array}{c} {\dot{x}}_{2}=x_{3}+f_{2}(x_{1},x_{2})+d_{2}(t), \end{array}$$ (20)

$$\begin{array}{c} {\dot{x}}_{3}=f_{3}(x_{2},x_{3})+g_{3}u+d_{3}(t), \end{array}$$ (21)

where $x=[x_{1},x_{2},x_{3}{]}^{T}\in {\mathbb{R}}^{3}$ is the state vector, u∈ℝ is the control input, and *y* = *x*_1_ is the system output. The functions $f_{i}(\cdot )$, *i* = 1,2,3, are unknown continuous nonlinearities representing the combined effects of unmodeled dynamics and parametric uncertainties. The constants *g*_1_ > 0 and *g*_3_ > 0 are unknown control gains, and $d_{i}(t)$, *i* = 1,2,3, denote unknown time-varying external disturbances.

### 2.3 Assumptions

To facilitate the control design and rigorous stability analysis, the following assumptions are imposed.

**Assumption 1:** All state variables $x_{1},x_{2},x_{3}$ are measurable and available for feedback. In a practical UAV system, the flight path angle *x*_1_ can be obtained from the inertial navigation system (INS) or GPS, the angle of attack *x*_2_ from an air data boom or angle-of-attack vane, and the pitch rate *x*_3_ from a rate gyroscope. This assumption is standard in output-feedback or full-state feedback control designs.

**Assumption 2:** There exist known positive constants $g_{1m},g_{1M},g_{3m},g_{3M}$ such that


$$\begin{array}{c} 0<g_{1m}\leq g_{1}\leq g_{1M},\;0<g_{3m}\leq g_{3}\leq g_{3M}. \end{array}$$ (22)

This assumption is physically motivated: the lift curve slope ${\bar{L}}_{\alpha }$ and the elevator effectiveness $M_{\delta }$ are positive and bounded for a conventional fixed-wing UAV. The bounds can be estimated from known aerodynamic data or identified from flight test data.

**Assumption 3:** The unknown nonlinear functions $f_{1}(x_{1})$, $f_{2}(x_{1},x_{2})$, and $f_{3}(x_{2},x_{3})$ are continuous on their respective domains. This is a mild assumption satisfied by most physical systems, including the UAV longitudinal dynamics, as the functions involve trigonometric and polynomial terms.

**Assumption 4:** The desired reference trajectory *x*_1*d*_(*t*) and its derivatives up to the second order are continuous and bounded. That is, there exists a positive constant *B*_0_ such that


$$\begin{array}{c} x_{1d}^{2}+{\dot{x}}_{1d}^{2}+{\ddot{x}}_{1d}^{2}\leq B_{0},\;\forall t\geq 0. \end{array}$$ (23)

This assumption ensures that the reference signal is smooth and does not contain discontinuities that would require infinite control effort. In practice, this can be satisfied by pre-filtering the reference commands.

**Assumption 5:** The external disturbances $d_{i}(t)$, *i* = 1,2,3, and their time derivatives ${\dot{d}}_{i}(t)$ are bounded, i.e., there exist known positive constants $\rho_{i}$ and $\nu_{i}$ such that


$$\begin{array}{c} |d_{i}(t)|\leq \rho_{i},\;|{\dot{d}}_{i}(t)|\leq \nu_{i},\;\forall t\geq 0. \end{array}$$ (24)

This assumption is reasonable for physical disturbances such as wind gusts, which have finite amplitude and rate of change. The bounds $\rho_{i}$ are used in the control design for robust compensation, while $\nu_{i}$ are used in the disturbance observer convergence analysis.

**Assumption 6:** The neural network approximation errors $\varepsilon_{i}(Z_{i})$ are bounded by unknown constants $\varepsilon_{iM}$, i.e.,


$$\begin{array}{c} |\varepsilon_{i}(Z_{i})|\leq \varepsilon_{iM},\;i=1,2,3, \end{array}$$ (25)

and the ideal weight vectors $W_{i}^{*}$ satisfy $\|W_{i}^{*}\|\leq W_{M}$ for some known $W_{M}>0$. This is a standard assumption in the adaptive neural control literature and is justified by the universal approximation property on compact sets.

### 2.4 Preliminaries on RBF neural networks

**Fig 2:**
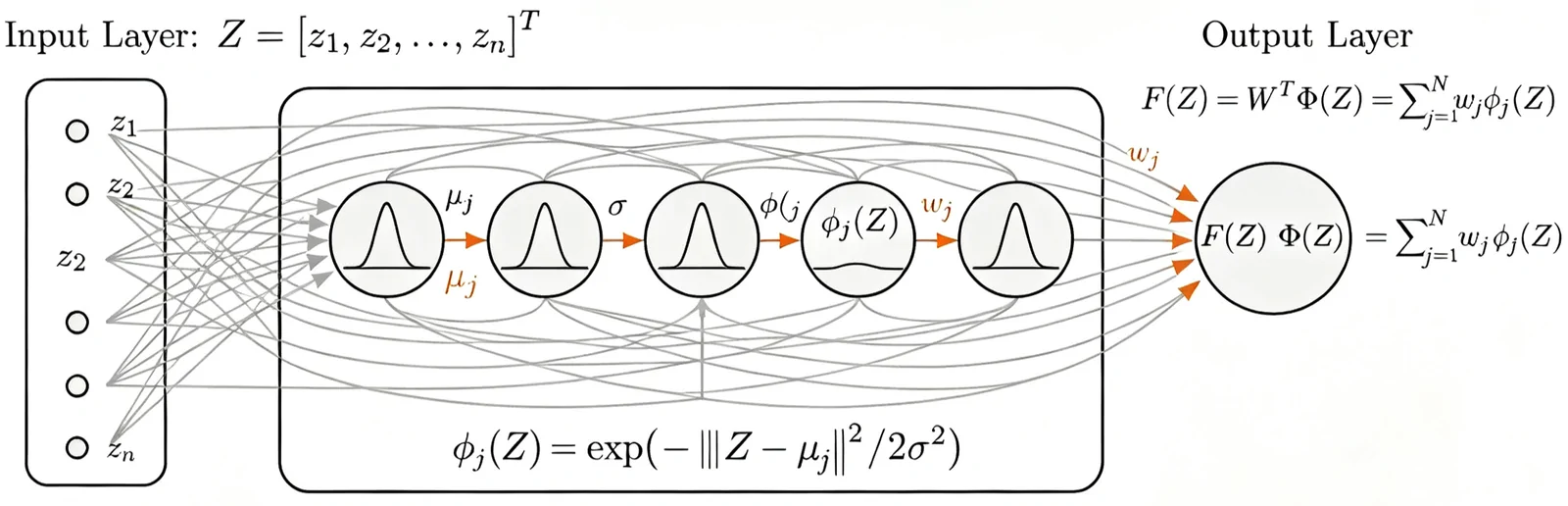
Architecture of the RBF neural network used for function approximation. The network has *n* inputs, *N* hidden neurons with Gaussian activation functions, and a single linear output.

Radial basis function (RBF) neural networks are employed in this paper to approximate the unknown continuous functions $f_{i}$ appearing in the system dynamics [43–45]. An RBF network is a two-layer structure consisting of a hidden layer with *N* nonlinear Gaussian basis functions and a linear output layer, as shown in Fig 2. For an input vector $Z\in \Omega_{Z}\subset {\mathbb{R}}^{n}$, the network output is given by:


$$\begin{array}{c} F(Z)=\sum_{j=1}^{N}w_{j}\phi_{j}(Z)=W^{T}\Phi (Z), \end{array}$$ (26)

where $W=[w_{1},...,w_{N}{]}^{T}\in {\mathbb{R}}^{N}$ is the weight vector, and $\Phi (Z)=[\phi_{1}(Z),...,\phi_{N}(Z){]}^{T}\in {\mathbb{R}}^{N}$ is the vector of basis functions. The Gaussian basis functions are defined as:


$$\begin{array}{c} \phi_{j}(Z)=\exp\left(-\frac{\|Z-\mu_{j}{\|}^{2}}{2\sigma^{2}}\right),\;j=1,...,N, \end{array}$$ (27)

where $\mu_{j}\in {\mathbb{R}}^{n}$ is the center of the *j*-th basis function, and σ>0 is the width parameter.

The universal approximation property of RBF networks states that for any continuous function $F(Z):\Omega_{Z}\to \mathbb{R}$ defined on a compact set $\Omega_{Z}\subset {\mathbb{R}}^{n}$, and for any desired accuracy $\varepsilon_{M}>0$, there exists an integer *N* and a weight vector $W^{*}\in {\mathbb{R}}^{N}$ such that:


$$\begin{array}{c} F(Z)=W^{*T}\Phi (Z)+\varepsilon (Z),\;|\varepsilon (Z)|\leq \varepsilon_{M},\;\forall Z\in \Omega_{Z}. \end{array}$$ (28)

The approximation error ε(Z) can be made arbitrarily small by increasing the number of neurons *N* and appropriately selecting the centers $\mu_{j}$ and width σ.

In this paper, we use three separate RBF networks to approximate the following lumped unknown functions:


$$\begin{array}{c} F_{1}(X_{1})=\frac{f_{1}(x_{1})}{g_{1}},\;X_{1}=x_{1}\in {\mathbb{R}}^{1}, \end{array}$$ (29)

$$\begin{array}{c} F_{2}(X_{2})=f_{2}(x_{1},x_{2}),\;X_{2}=[x_{1},x_{2}{]}^{T}\in {\mathbb{R}}^{2}, \end{array}$$ (30)

$$\begin{array}{c} F_{3}(X_{3})=\frac{f_{3}(x_{2},x_{3})}{g_{3}},\;X_{3}=[x_{2},x_{3}{]}^{T}\in {\mathbb{R}}^{2}. \end{array}$$ (31)

The lumped approximation strategy in (29) and (31) avoids the need to estimate *g*_1_ and *g*_3_ separately, as the network outputs combine directly with the virtual and actual control signals. Consequently, we have:


$$\begin{array}{c} \frac{f_{1}(x_{1})}{g_{1}}=W_{1}^{*T}\Phi_{1}(x_{1})+\varepsilon_{1},\;|\varepsilon_{1}|\leq \varepsilon_{1M}, \end{array}$$ (32)

$$\begin{array}{c} f_{2}(x_{1},x_{2})=W_{2}^{*T}\Phi_{2}(x_{1},x_{2})+\varepsilon_{2},\;|\varepsilon_{2}|\leq \varepsilon_{2M}, \end{array}$$ (33)

$$\begin{array}{c} \frac{f_{3}(x_{2},x_{3})}{g_{3}}=W_{3}^{*T}\Phi_{3}(x_{2},x_{3})+\varepsilon_{3},\;|\varepsilon_{3}|\leq \varepsilon_{3M}. \end{array}$$ (34)

The ideal weights $W_{i}^{*}$ are unknown and will be estimated online via adaptive laws to be designed later. The estimates are denoted as ${\hat{W}}_{i}$, and the estimation errors are defined as ${\tilde{W}}_{i}={\hat{W}}_{i}-W_{i}^{*}$.

**Remark 4:** The RBF networks employed in this work are configured with Gaussian basis functions. The centers $\mu_{j}$ are selected to cover the expected operating range of the input variables, determined from the UAV’s flight envelope, while the width σ governs the smoothness–accuracy trade-off: larger σ enhances generalization at the cost of approximation precision, whereas smaller σ improves local accuracy but risks overfitting. The universal approximation property of RBF networks is guaranteed on compact sets. The subsequent Lyapunov-based stability analysis establishes semi-global uniform ultimate boundedness of all closed-loop signals, ensuring that the system states remain within a compact region after the transient phase. This a posteriori justification legitimizes the use of the universal approximation property in the stability proof.

**Remark 5:** The RBF networks and disturbance observers serve distinct and complementary roles. The RBF networks approximate only the state-dependent unknown functions $f_{i}(\cdot )$ arising from structural uncertainties, while the disturbance observers estimate only the external time-varying disturbances $d_{i}(t)$, which are independent of system states. The RBF provides feedforward compensation for system nonlinearities, whereas the DOB ensures active rejection of external perturbations. If the RBF were tasked with approximating the lumped term $f_{i}+d_{i}$, the DOB would be rendered redundant. Our explicit separation of these two tasks preserves the effectiveness of both components without mutual interference.

## 3 Disturbance observer and adaptive neural DSC design

**Fig 3:**
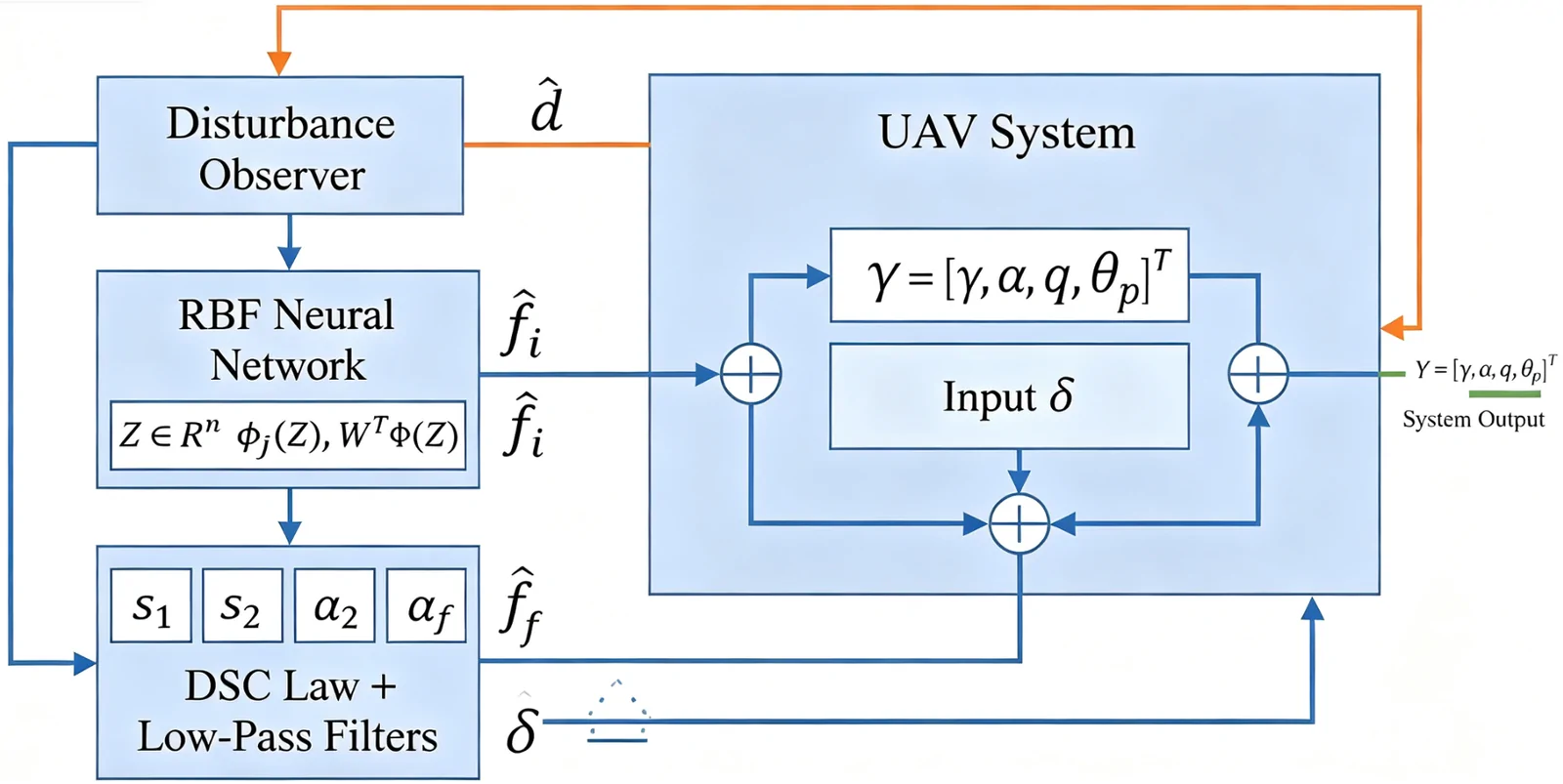
Overall architecture of the proposed disturbance observer based adaptive neural dynamic surface control scheme for fixed-wing UAV longitudinal motion.

In this section, we present the systematic design procedure of the proposed disturbance-observer-based adaptive neural dynamic surface control scheme. The design consists of three main components: (i) nonlinear disturbance observers for estimating unknown external disturbances, (ii) RBF neural networks for approximating unknown nonlinear functions, and (iii) dynamic surface control with first-order low-pass filters to avoid the explosion of complexity. The overall control architecture is illustrated in Fig 3, which shows the interconnection among the disturbance observers, the neural network approximators, the DSC filters, and the UAV system.

### 3.1 Nonlinear disturbance observer design

We begin by designing nonlinear disturbance observers to estimate the unknown external disturbances $d_{i}(t)$, *i* = 1,2,3. The observers are constructed based only on the available system states, without requiring knowledge of the disturbances or their derivatives.

For each subsystem, the disturbance observer is designed in the following form:


$$\begin{array}{c} {\hat{d}}_{i}=z_{i}+p_{i}(x_{i}), \end{array}$$ (35)

$$\begin{array}{c} {\dot{z}}_{i}=-l_{i}(x_{i})z_{i}-l_{i}(x_{i})(p_{i}(x_{i})+\phi_{i}(x)), \end{array}$$ (36)

where ${\hat{d}}_{i}$ is the estimate of $d_{i}$, $z_{i}$ is the internal state of the observer, $p_{i}(x_{i})$ is a smooth design function to be specified, $l_{i}(x_{i})=\partial p_{i}(x_{i})/\partial x_{i}$ is the observer gain, and $\phi_{i}(x)$ represents the known part of the dynamics in the *i*-th subsystem.

To simplify the observer design and analysis, we choose a linear structure for $p_{i}(x_{i})$:


$$\begin{array}{c} p_{i}(x_{i})=l_{i}x_{i},\;l_{i}>0, \end{array}$$ (37)

where $l_{i}$ is a positive constant to be designed. Consequently, the observer gain is also constant: $l_{i}(x_{i})=l_{i}$. With this choice, the observer dynamics (36) become:


$$\begin{array}{c} {\dot{z}}_{i}=-l_{i}z_{i}-l_{i}(l_{i}x_{i}+\phi_{i}(x)). \end{array}$$ (38)

The disturbance estimate is then given by:


$$\begin{array}{c} {\hat{d}}_{i}=z_{i}+l_{i}x_{i}. \end{array}$$ (39)

Differentiating ${\hat{d}}_{i}$ with respect to time yields:


$$\begin{array}{c} \begin{array}{cc} {\dot{\hat{d}}}_{i} & ={\dot{z}}_{i}+l_{i}{\dot{x}}_{i}\\ & =-l_{i}z_{i}-l_{i}(l_{i}x_{i}+\phi_{i})+l_{i}(\phi_{i}+d_{i})\\ & =-l_{i}(z_{i}+l_{i}x_{i})+l_{i}d_{i}\\ & =-l_{i}{\hat{d}}_{i}+l_{i}d_{i}. \end{array} \end{array}$$ (40)

Define the disturbance estimation error as:


$$\begin{array}{c} {\tilde{d}}_{i}=d_{i}-{\hat{d}}_{i}. \end{array}$$ (41)

Subtracting (40) from the derivative of $d_{i}$, we obtain the error dynamics:


$$\begin{array}{c} \begin{array}{cc} {\dot{\tilde{d}}}_{i} & ={\dot{d}}_{i}-{\dot{\hat{d}}}_{i}={\dot{d}}_{i}-(-l_{i}{\hat{d}}_{i}+l_{i}d_{i})\\ & =-l_{i}{\tilde{d}}_{i}+{\dot{d}}_{i}. \end{array} \end{array}$$ (42)

The differential equation (42) is a first-order linear system driven by ${\dot{d}}_{i}$. Its solution can be expressed as:


$$\begin{array}{c} {\tilde{d}}_{i}(t)=e^{-l_{i}t}{\tilde{d}}_{i}(0)+\int_{0}^{t}e^{-l_{i}(t-\tau )}{\dot{d}}_{i}(\tau )\;d\tau . \end{array}$$ (43)

Under Assumption 5, $|{\dot{d}}_{i}(t)|\leq \nu_{i}$, we can bound the estimation error as:


$$\begin{array}{c} |{\tilde{d}}_{i}(t)|\leq e^{-l_{i}t}|{\tilde{d}}_{i}(0)|+\frac{\nu_{i}}{l_{i}}(1-e^{-l_{i}t})\leq e^{-l_{i}t}|{\tilde{d}}_{i}(0)|+\frac{\nu_{i}}{l_{i}}. \end{array}$$ (44)

Thus, by choosing the observer gain $l_{i}$ sufficiently large, the steady-state estimation error can be made arbitrarily small. Moreover, the convergence rate is exponential with decay rate $l_{i}$. For the subsequent stability analysis, we assume there exists a known bound $\delta_{i}>0$ such that:


$$\begin{array}{c} |{\tilde{d}}_{i}(t)|\leq \delta_{i},\;\forall t\geq 0. \end{array}$$ (45)

**Remark 6:** The disturbance observer design presented above is model-free in the sense that it does not require explicit knowledge of the disturbance dynamics. The only requirement is the boundedness of ${\dot{d}}_{i}$, which is satisfied for most physical disturbances. The observer gain $l_{i}$ serves as a tuning parameter that directly influences the estimation speed and accuracy: larger $l_{i}$ yields faster convergence but may amplify measurement noise.

**Remark 7:** For the UAV application, the disturbances $d_{1},d_{2},d_{3}$ correspond to wind gust components affecting the flight path angle, angle of attack, and pitch rate channels, respectively. These disturbances are typically band-limited and have bounded derivatives, making the proposed observer well-suited for this application.

### 3.2 Adaptive neural DSC design with disturbance compensation

With the disturbance observers in place, we now proceed to design the adaptive neural dynamic surface control law. The design follows a three-step recursive procedure, where at each step we introduce a virtual control law, an RBF neural network for function approximation, an adaptive law for weight updating, and a first-order low-pass filter to generate the next virtual control signal.

#### 3.2.1 Step 1: Virtual control for *x*_2_.

Define the first tracking error surface:


$$\begin{array}{c} S_{1}=x_{1}-x_{1d}. \end{array}$$ (46)

Differentiating *S*_1_ along the system dynamics (19) yields:


$$\begin{array}{c} {\dot{S}}_{1}=g_{1}x_{2}+f_{1}(x_{1})+d_{1}-{\dot{x}}_{1d}. \end{array}$$ (47)

Since *g*_1_ and $f_{1}(x_{1})$ are unknown, we define the lumped unknown function:


$$\begin{array}{c} F_{1}(X_{1})=\frac{f_{1}(x_{1})}{g_{1}},\;X_{1}=x_{1}. \end{array}$$ (48)

Using the RBF neural network approximation property, we have:


$$\begin{array}{c} F_{1}(X_{1})=W_{1}^{*T}\Phi_{1}(X_{1})+\varepsilon_{1},\;|\varepsilon_{1}|\leq \varepsilon_{1M}, \end{array}$$ (49)

where $W_{1}^{*}$ is the ideal weight vector, $\Phi_{1}(X_{1})$ is the basis function vector, and $\varepsilon_{1}$ is the approximation error.

Substituting (49) into (47), we obtain:


$$\begin{array}{c} {\dot{S}}_{1}=g_{1}\left(x_{2}+W_{1}^{*T}\Phi_{1}+\varepsilon_{1}\right)+d_{1}-{\dot{x}}_{1d}. \end{array}$$ (50)

We now design the virtual control ${\bar{x}}_{2}$ as follows:


$$\begin{array}{c} {\bar{x}}_{2}=-{\hat{W}}_{1}^{T}\Phi_{1}-\frac{\rho_{1}^{2}S_{1}}{2\epsilon }-c_{1}S_{1}-\frac{{\hat{d}}_{1}}{g_{1m}}, \end{array}$$ (51)

where ${\hat{W}}_{1}$ is the estimate of $W_{1}^{*}$, *c*_1_ > 0 is a design parameter, ϵ>0 is a small constant for the nonlinear damping term, and ${\hat{d}}_{1}$ is the disturbance estimate from the DOB. The term $-\rho_{1}^{2}S_{1}/(2\epsilon )$ serves as a nonlinear damping term to compensate for the disturbance estimation error and approximation error, while $-{\hat{d}}_{1}/g_{1m}$ provides active disturbance compensation.

Define the weight estimation error as:


$$\begin{array}{c} {\tilde{W}}_{1}={\hat{W}}_{1}-W_{1}^{*}. \end{array}$$ (52)

Let the filter error be defined as:


$$\begin{array}{c} y_{2}=x_{2d}-{\bar{x}}_{2}, \end{array}$$ (53)

where *x*_2*d*_ is the filtered signal obtained by passing ${\bar{x}}_{2}$ through a first-order low-pass filter:


$$\begin{array}{c} \tau_{2}{\dot{x}}_{2d}+x_{2d}={\bar{x}}_{2},\;x_{2d}(0)={\bar{x}}_{2}(0). \end{array}$$ (54)

Then, we have $x_{2}=S_{2}+x_{2d}=S_{2}+y_{2}+{\bar{x}}_{2}$, where $S_{2}=x_{2}-x_{2d}$ is the second error surface to be defined later. Substituting this relation and the virtual control (51) into (50), we obtain:


$$\begin{array}{c} \begin{array}{cc} {\dot{S}}_{1} & =g_{1}\left(S_{2}+y_{2}+{\bar{x}}_{2}+W_{1}^{*T}\Phi_{1}+\varepsilon_{1}\right)+d_{1}-{\dot{x}}_{1d}\\ & =g_{1}\left(S_{2}+y_{2}-{\tilde{W}}_{1}^{T}\Phi_{1}+\varepsilon_{1}\right)-\frac{g_{1}}{g_{1m}}{\hat{d}}_{1}+d_{1}-\frac{\rho_{1}^{2}S_{1}}{2\epsilon }-c_{1}S_{1}\\ & =g_{1}(S_{2}+y_{2}-{\tilde{W}}_{1}^{T}\Phi_{1}+\varepsilon_{1})+{\tilde{d}}_{1}-\left(1-\frac{g_{1}}{g_{1m}}\right){\hat{d}}_{1}-\frac{\rho_{1}^{2}S_{1}}{2\epsilon }-c_{1}S_{1}. \end{array} \end{array}$$ (55)

Since $g_{1}\leq g_{1m}$ by Assumption 2, we have $0\leq 1-g_{1}/g_{1m}<1$. The term ${\tilde{d}}_{1}-(1-g_{1}/g_{1m}){\hat{d}}_{1}$ can be bounded using the disturbance estimation error bound and the disturbance bound. Specifically:


$$\begin{array}{c} \left|{\tilde{d}}_{1}-\left(1-\frac{g_{1}}{g_{1m}}\right){\hat{d}}_{1}\right|\leq |{\tilde{d}}_{1}|+\left|1-\frac{g_{1}}{g_{1m}}\right||{\hat{d}}_{1}|\leq \delta_{1}+\rho_{1}={\bar{\rho }}_{1}, \end{array}$$ (56)

where ${\bar{\rho }}_{1}=\delta_{1}+\rho_{1}$. Thus, the nonlinear damping term $-\rho_{1}^{2}S_{1}/(2\epsilon )$ will be sufficient to dominate the combined disturbance effect if we replace $\rho_{1}$ with ${\bar{\rho }}_{1}$ in the design. In practice, since $\delta_{1}$ can be made small by choosing large *l*_1_, we retain $\rho_{1}$ as the design parameter for simplicity, with the understanding that the stability proof will account for the additional bounded residual.

The adaptive law for ${\hat{W}}_{1}$ is designed as:


$$\begin{array}{c} {\dot{\hat{W}}}_{1}=\Gamma_{1}\Phi_{1}S_{1}-\Gamma_{1}\eta_{1}{\hat{W}}_{1}, \end{array}$$ (57)

where $\Gamma_{1}=\Gamma_{1}^{T}>0$ is the adaptation gain matrix, and $\eta_{1}>0$ is the σ-modification coefficient. The σ-modification term $-\Gamma_{1}\eta_{1}{\hat{W}}_{1}$ is introduced to prevent parameter drift and ensure boundedness of the weight estimates in the presence of approximation errors and disturbances.

**Remark 8:** The virtual control (51) contains three key components: (i) $-{\hat{W}}_{1}^{T}\Phi_{1}$ for feedforward compensation of the unknown dynamics, (ii) $-\rho_{1}^{2}S_{1}/(2\epsilon )$ for robust damping against residual errors, and (iii) $-{\hat{d}}_{1}/g_{1m}$ for active disturbance compensation.

#### 3.2.2 Step 2: Virtual control for *x*_3_.

Define the second tracking error surface:


$$\begin{array}{c} S_{2}=x_{2}-x_{2d}. \end{array}$$ (58)

Differentiating *S*_2_ along (20) gives:


$$\begin{array}{c} {\dot{S}}_{2}=x_{3}+f_{2}(x_{1},x_{2})+d_{2}-{\dot{x}}_{2d}. \end{array}$$ (59)

The unknown function $f_{2}(x_{1},x_{2})$ is approximated by the second RBF network:


$$\begin{array}{c} f_{2}(x_{1},x_{2})=W_{2}^{*T}\Phi_{2}(X_{2})+\varepsilon_{2},\;|\varepsilon_{2}|\leq \varepsilon_{2M}, \end{array}$$ (60)

where $X_{2}=[x_{1},x_{2}{]}^{T}$.

We design the virtual control ${\bar{x}}_{3}$ as:


$$\begin{array}{c} {\bar{x}}_{3}=-{\hat{W}}_{2}^{T}\Phi_{2}-\frac{\rho_{2}^{2}S_{2}}{2\epsilon }-c_{2}S_{2}+{\dot{x}}_{2d}-{\hat{d}}_{2}, \end{array}$$ (61)

where *c*_2_ > 0 is a design parameter, and ${\hat{d}}_{2}$ is the disturbance estimate from the second DOB.

Define ${\tilde{W}}_{2}={\hat{W}}_{2}-W_{2}^{*}$ and $y_{3}=x_{3d}-{\bar{x}}_{3}$, where *x*_3*d*_ is obtained from:


$$\begin{array}{c} \tau_{3}{\dot{x}}_{3d}+x_{3d}={\bar{x}}_{3},\;x_{3d}(0)={\bar{x}}_{3}(0). \end{array}$$ (62)

Using $x_{3}=S_{3}+x_{3d}=S_{3}+y_{3}+{\bar{x}}_{3}$ and substituting (61) into (59), we obtain the closed-loop error dynamics:


$$\begin{array}{c} \begin{array}{cc} {\dot{S}}_{2} & =S_{3}+y_{3}-{\tilde{W}}_{2}^{T}\Phi_{2}+\varepsilon_{2}+d_{2}-{\hat{d}}_{2}-\frac{\rho_{2}^{2}S_{2}}{2\epsilon }-c_{2}S_{2}\\ & =S_{3}+y_{3}-{\tilde{W}}_{2}^{T}\Phi_{2}+\varepsilon_{2}+{\tilde{d}}_{2}-\frac{\rho_{2}^{2}S_{2}}{2\epsilon }-c_{2}S_{2}. \end{array} \end{array}$$ (63)

The adaptive law for ${\hat{W}}_{2}$ is designed as:


$$\begin{array}{c} {\dot{\hat{W}}}_{2}=\Gamma_{2}\Phi_{2}S_{2}-\Gamma_{2}\eta_{2}{\hat{W}}_{2}, \end{array}$$ (64)

where $\Gamma_{2}=\Gamma_{2}^{T}>0$ and $\eta_{2}>0$.

#### 3.2.3 Step 3: Actual control law.

Define the third tracking error surface:


$$\begin{array}{c} S_{3}=x_{3}-x_{3d}. \end{array}$$ (65)

Differentiating *S*_3_ along (21) yields:


$$\begin{array}{c} {\dot{S}}_{3}=f_{3}(x_{2},x_{3})+g_{3}u+d_{3}-{\dot{x}}_{3d}. \end{array}$$ (66)

Define the lumped unknown function:


$$\begin{array}{c} F_{3}(X_{3})=\frac{f_{3}(x_{2},x_{3})}{g_{3}},\;X_{3}=[x_{2},x_{3}{]}^{T}. \end{array}$$ (67)

The third RBF network provides the approximation:


$$\begin{array}{c} F_{3}(X_{3})=W_{3}^{*T}\Phi_{3}(X_{3})+\varepsilon_{3},\;|\varepsilon_{3}|\leq \varepsilon_{3M}. \end{array}$$ (68)

The actual control law is designed as:


$$\begin{array}{c} u=-{\hat{W}}_{3}^{T}\Phi_{3}-\frac{\rho_{3}^{2}S_{3}}{2\epsilon }-c_{3}S_{3}-\frac{{\hat{d}}_{3}}{g_{3m}}, \end{array}$$ (69)

where *c*_3_ > 0 is a design parameter, ${\hat{W}}_{3}$ is the estimate of $W_{3}^{*}$, and ${\hat{d}}_{3}$ is the disturbance estimate from the third DOB.

Define ${\tilde{W}}_{3}={\hat{W}}_{3}-W_{3}^{*}$. Substituting (69) into (66) yields the closed-loop error dynamics:


$$\begin{array}{c} \begin{array}{cc} {\dot{S}}_{3} & =g_{3}\left(-{\hat{W}}_{3}^{T}\Phi_{3}-\frac{\rho_{3}^{2}S_{3}}{2\epsilon }-c_{3}S_{3}-\frac{{\hat{d}}_{3}}{g_{3m}}+W_{3}^{*T}\Phi_{3}+\varepsilon_{3}\right)+d_{3}-{\dot{x}}_{3d}\\ & =-g_{3}{\tilde{W}}_{3}^{T}\Phi_{3}+g_{3}\varepsilon_{3}-\frac{g_{3}}{g_{3m}}{\hat{d}}_{3}+d_{3}-\frac{\rho_{3}^{2}S_{3}}{2\epsilon }-c_{3}S_{3}\\ & =-g_{3}{\tilde{W}}_{3}^{T}\Phi_{3}+g_{3}\varepsilon_{3}+{\tilde{d}}_{3}-\left(1-\frac{g_{3}}{g_{3m}}\right){\hat{d}}_{3}-\frac{\rho_{3}^{2}S_{3}}{2\epsilon }-c_{3}S_{3}. \end{array} \end{array}$$ (70)

The adaptive law for ${\hat{W}}_{3}$ is designed as:


$$\begin{array}{c} {\dot{\hat{W}}}_{3}=\Gamma_{3}\Phi_{3}S_{3}-\Gamma_{3}\eta_{3}{\hat{W}}_{3}, \end{array}$$ (71)

where $\Gamma_{3}=\Gamma_{3}^{T}>0$ and $\eta_{3}>0$.

### 3.3 Filter error dynamics

The filter errors *y*_2_ and *y*_3_ defined in (53) and similarly for *y*_3_ play a crucial role in the stability analysis. Their dynamics are derived from the filter equations (54) and (62).

From (54), we have:


$$\begin{array}{c} \tau_{2}{\dot{x}}_{2d}={\bar{x}}_{2}-x_{2d}=-y_{2}. \end{array}$$ (72)

Thus:


$$\begin{array}{c} {\dot{x}}_{2d}=-\frac{y_{2}}{\tau_{2}}. \end{array}$$ (73)

Differentiating $y_{2}=x_{2d}-{\bar{x}}_{2}$ with respect to time:


$$\begin{array}{c} \begin{array}{cc} {\dot{y}}_{2} & ={\dot{x}}_{2d}-{\dot{\bar{x}}}_{2}\\ & =-\frac{y_{2}}{\tau_{2}}-{\dot{\bar{x}}}_{2}. \end{array} \end{array}$$ (74)

Similarly, for $y_{3}=x_{3d}-{\bar{x}}_{3}$:


$$\begin{array}{c} \begin{array}{cc} {\dot{y}}_{3} & ={\dot{x}}_{3d}-{\dot{\bar{x}}}_{3}\\ & =-\frac{y_{3}}{\tau_{3}}-{\dot{\bar{x}}}_{3}. \end{array} \end{array}$$ (75)

**Remark 9:** The bounding functions *B*_2_ and *B*_3_ are continuous on compact sets, depending continuously on the system signals. The semi-global nature of the stability analysis arises from their boundedness on any compact region of initial conditions. Furthermore, the disturbance compensation terms do not increase filter design complexity, as the filters operate on composite virtual signals and the DOB estimates ${\hat{d}}_{i}$ are inherently smooth.

### 3.4 Algorithm summary

To facilitate practical implementation and enhance the reproducibility of the proposed control scheme, the complete design procedure is summarized in Algorithm 1. The algorithm executes in real time at each sampling instant, sequentially performing disturbance observer updates, error surface computations, neural network adaptations, virtual/actual control law calculations, and first-order filter updates.

**Algorithm 1 Disturbance-Observer-Based Adaptive Neural DSC.**

**Require:** System states $x_{1},x_{2},x_{3}$; reference signal *x*_1*d*_ and its derivatives ${\dot{x}}_{1d},{\ddot{x}}_{1d}$; design parameters $c_{i},\eta_{i},\Gamma_{i},\tau_{i},\epsilon ,\rho_{i},l_{i},g_{1m},g_{3m}$; RBF network parameters $\Phi_{i}(\cdot )$

**Ensure:** Control input *u*

1:  **Initialization:** Set ${\hat{W}}_{1}(0)=0$, ${\hat{W}}_{2}(0)=0$, ${\hat{W}}_{3}(0)=0$, *z*_1_(0)=0, *z*_2_(0)=0, *z*_3_(0)=0, $x_{2d}(0)={\bar{x}}_{2}(0)$, $x_{3d}(0)={\bar{x}}_{3}(0)$

2:  **for** each sampling instant **do**

3:   **Step 1: Disturbance Observer Updates**

4:   ${\hat{d}}_{i}=z_{i}+l_{i}x_{i},\;i=1,2,3$

5:   ${\dot{z}}_{i}=-l_{i}z_{i}-l_{i}(l_{i}x_{i}+\phi_{i}(x)),\;i=1,2,3$

6:   **Step 2: Error Surface Computations**

7:   $S_{1}=x_{1}-x_{1d}$

8:   $S_{2}=x_{2}-x_{2d}$

9:   $S_{3}=x_{3}-x_{3d}$

10:   **Step 3: Neural Network Adaptation**

11:   ${\dot{\hat{W}}}_{1}=\Gamma_{1}(\Phi_{1}S_{1}-\eta_{1}{\hat{W}}_{1})$

12:   ${\dot{\hat{W}}}_{2}=\Gamma_{2}(\Phi_{2}S_{2}-\eta_{2}{\hat{W}}_{2})$

13:   ${\dot{\hat{W}}}_{3}=\Gamma_{3}(\Phi_{3}S_{3}-\eta_{3}{\hat{W}}_{3})$

14:   **Step 4: Virtual and Actual Control Laws**

15:   ${\bar{x}}_{2}=-{\hat{W}}_{1}^{T}\Phi_{1}-\frac{\rho_{1}^{2}S_{1}}{2\epsilon }-c_{1}S_{1}-\frac{{\hat{d}}_{1}}{g_{1m}}$

16:   ${\bar{x}}_{3}=-{\hat{W}}_{2}^{T}\Phi_{2}-\frac{\rho_{2}^{2}S_{2}}{2\epsilon }-c_{2}S_{2}+{\dot{x}}_{2d}-{\hat{d}}_{2}$

17:   $u=-{\hat{W}}_{3}^{T}\Phi_{3}-\frac{\rho_{3}^{2}S_{3}}{2\epsilon }-c_{3}S_{3}-\frac{{\hat{d}}_{3}}{g_{3m}}$

18:   **Step 5: First-Order Filter Updates**

19:   $\tau_{2}{\dot{x}}_{2d}+x_{2d}={\bar{x}}_{2}$

20:   $\tau_{3}{\dot{x}}_{3d}+x_{3d}={\bar{x}}_{3}$

21:  **end for**

22:  **Output:** Apply *u* to the UAV system

### 3.5 Stability analysis

In this section, we present the rigorous Lyapunov-based stability analysis of the closed-loop system. The analysis establishes the semi-global uniform ultimate boundedness (SGUUB) of all closed-loop signals, including the tracking errors $S_{i}$, filter errors $y_{i}$, weight estimation errors ${\tilde{W}}_{i}$, and disturbance estimation errors ${\tilde{d}}_{i}$. The proof follows a systematic procedure: we first define the composite Lyapunov function candidate, then derive its time derivative along the closed-loop trajectories, and finally apply standard Lyapunov arguments with appropriate bounding techniques.

#### 3.5.1 Preliminary Lemmas.

Before proceeding to the main theorem, we introduce the following lemma, which is essential for handling the filter error dynamics in the stability proof.

**Lemma 1:** Consider the filter errors $y_{2}=x_{2d}-{\bar{x}}_{2}$ and $y_{3}=x_{3d}-{\bar{x}}_{3}$, where ${\bar{x}}_{2}$ and ${\bar{x}}_{3}$ are the virtual control laws defined in (51) and (61), and *x*_2*d*_, *x*_3*d*_ are the filtered signals from (54) and (62). Then, there exist nonnegative continuous functions *B*_2_ and *B*_3_ such that


$$\begin{array}{c} \left|{\dot{y}}_{2}+\frac{y_{2}}{\tau_{2}}\right|\leq B_{2}(S_{1},S_{2},y_{2},{\tilde{W}}_{1},{\tilde{d}}_{1},x_{1d},{\dot{x}}_{1d},{\ddot{x}}_{1d}), \end{array}$$ (76)

$$\begin{array}{c} \left|{\dot{y}}_{3}+\frac{y_{3}}{\tau_{3}}\right|\leq B_{3}(S_{1},S_{2},S_{3},y_{2},y_{3},{\tilde{W}}_{1},{\tilde{W}}_{2},{\tilde{d}}_{1},{\tilde{d}}_{2},x_{1d},{\dot{x}}_{1d},{\ddot{x}}_{1d}). \end{array}$$ (77)

Moreover, for any given positive constant *p*, the functions *B*_2_ and *B*_3_ are bounded on the compact set


$$\begin{array}{c} \Omega =\left\{\sum_{i=1}^{3}S_{i}^{2}+y_{2}^{2}+y_{3}^{2}+\sum_{i=1}^{3}{\tilde{W}}_{i}^{T}\Gamma_{i}^{-1}{\tilde{W}}_{i}+\sum_{i=1}^{3}{\tilde{d}}_{i}^{2}\leq 2p\right\}. \end{array}$$ (78)

The virtual control laws ${\bar{x}}_{2}$ and ${\bar{x}}_{3}$ are continuously differentiable functions of their arguments. Specifically, ${\bar{x}}_{2}$ depends on *S*_1_, ${\hat{W}}_{1}$, $\Phi_{1}$, ${\hat{d}}_{1}$, and *x*_1*d*_, while ${\bar{x}}_{3}$ depends on *S*_2_, ${\hat{W}}_{2}$, $\Phi_{2}$, ${\hat{d}}_{2}$, ${\dot{x}}_{2d}$, and *x*_2*d*_. Their time derivatives involve the derivatives of these arguments, which are all continuous functions of the system states and reference signals. Since the reference signal *x*_1*d*_ and its derivatives up to second order are bounded by Assumption 4, and since all signals remain within the compact set Ω, the functions *B*_2_ and *B*_3_ are continuous and hence bounded on Ω. This completes the proof.

#### 3.5.2 Main stability theorem.

**Theorem:** Consider the uncertain strict-feedback nonlinear system (19)–(21) satisfying Assumptions 1–6. Apply the disturbance observers (35)–(39), the virtual control laws (51)–(61), the actual control law (69), and the adaptive laws (57)–(71). Then, for any bounded initial conditions and any given compact set, there exist design parameters $c_{i}$, $\eta_{i}$, $\Gamma_{i}$, $\tau_{i}$, ϵ, $\rho_{i}$, and $l_{i}$ such that all closed-loop signals are semi-globally uniformly ultimately bounded (SGUUB). Moreover, the tracking error $S_{1}=x_{1}-x_{1d}$ can be made arbitrarily small by choosing the design parameters appropriately.

**Proof:** We construct the following composite Lyapunov function candidate as


$$\begin{array}{c} V=V_{S}+V_{y}+V_{W}+V_{d}, \end{array}$$ (79)

where


$$\begin{array}{c} V_{S}=\frac{1}{2}\sum_{i=1}^{3}S_{i}^{2}, \end{array}$$ (80)

$$\begin{array}{c} V_{y}=\frac{1}{2}(y_{2}^{2}+y_{3}^{2}), \end{array}$$ (81)

$$\begin{array}{c} V_{W}=\frac{1}{2}\sum_{i=1}^{3}{\tilde{W}}_{i}^{T}\Gamma_{i}^{-1}{\tilde{W}}_{i}, \end{array}$$ (82)

$$\begin{array}{c} V_{d}=\frac{1}{2}\sum_{i=1}^{3}{\tilde{d}}_{i}^{2}. \end{array}$$ (83)

**Step 1: Derivative of** $V_{S}$.

Differentiating $V_{S}$ along the closed-loop error dynamics (55), (63), and (70) yields:


$$\begin{array}{c} \begin{array}{cc} {\dot{V}}_{S} & =S_{1}{\dot{S}}_{1}+S_{2}{\dot{S}}_{2}+S_{3}{\dot{S}}_{3}\\ & =-c_{1}S_{1}^{2}-c_{2}S_{2}^{2}-c_{3}S_{3}^{2}-\frac{\rho_{1}^{2}S_{1}^{2}}{2\epsilon }-\frac{\rho_{2}^{2}S_{2}^{2}}{2\epsilon }-\frac{\rho_{3}^{2}S_{3}^{2}}{2\epsilon }\\ & \;+g_{1}S_{1}S_{2}+g_{1}S_{1}y_{2}-g_{1}S_{1}{\tilde{W}}_{1}^{T}\Phi_{1}+g_{1}S_{1}\varepsilon_{1}\\ & \;+S_{2}S_{3}+S_{2}y_{3}-S_{2}{\tilde{W}}_{2}^{T}\Phi_{2}+S_{2}\varepsilon_{2}+S_{2}{\tilde{d}}_{2}\\ & \;-g_{3}S_{3}{\tilde{W}}_{3}^{T}\Phi_{3}+g_{3}S_{3}\varepsilon_{3}+S_{3}{\tilde{d}}_{3}\\ & \;+S_{1}{\tilde{d}}_{1}-S_{1}\left(1-\frac{g_{1}}{g_{1m}}\right){\hat{d}}_{1}-S_{3}\left(1-\frac{g_{3}}{g_{3m}}\right){\hat{d}}_{3}. \end{array} \end{array}$$ (84)

Applying Young’s inequality and Assumption 2 ($g_{1}\leq g_{1m}$, $g_{3}\leq g_{3m}$), the disturbance-related terms can be bounded as:


$$\begin{array}{c} |S_{1}{\tilde{d}}_{1}|\leq \frac{\rho_{1}^{2}S_{1}^{2}}{2\epsilon }+\frac{\epsilon }{2}, \end{array}$$ (85)

$$\begin{array}{c} |S_{2}{\tilde{d}}_{2}|\leq \frac{\rho_{2}^{2}S_{2}^{2}}{2\epsilon }+\frac{\epsilon }{2}, \end{array}$$ (86)

$$\begin{array}{c} |S_{3}{\tilde{d}}_{3}|\leq \frac{\rho_{3}^{2}S_{3}^{2}}{2\epsilon }+\frac{\epsilon }{2}, \end{array}$$ (87)

where we have used $|{\tilde{d}}_{i}|\leq \rho_{i}$ from Assumption 5 and $\delta_{i}\leq \rho_{i}$ for simplicity.

The terms involving ${\hat{d}}_{i}$ are bounded as:


$$\begin{array}{c} \left|S_{1}\left(1-\frac{g_{1}}{g_{1m}}\right){\hat{d}}_{1}\right|\leq \frac{\rho_{1}^{2}S_{1}^{2}}{2\epsilon }+\frac{\epsilon }{2}, \end{array}$$ (88)

$$\begin{array}{c} \left|S_{3}\left(1-\frac{g_{3}}{g_{3m}}\right){\hat{d}}_{3}\right|\leq \frac{\rho_{3}^{2}S_{3}^{2}}{2\epsilon }+\frac{\epsilon }{2}. \end{array}$$ (89)

The neural approximation error terms are bounded using Young’s inequality:


$$\begin{array}{c} g_{1}S_{1}\varepsilon_{1}\leq \frac{\rho_{1}^{2}S_{1}^{2}}{2\epsilon }+\frac{g_{1}^{2}\epsilon \varepsilon_{1M}^{2}}{2\rho_{1}^{2}}, \end{array}$$ (90)

$$\begin{array}{c} S_{2}\varepsilon_{2}\leq \frac{\rho_{2}^{2}S_{2}^{2}}{2\epsilon }+\frac{\epsilon \varepsilon_{2M}^{2}}{2\rho_{2}^{2}}, \end{array}$$ (91)

$$\begin{array}{c} g_{3}S_{3}\varepsilon_{3}\leq \frac{\rho_{3}^{2}S_{3}^{2}}{2\epsilon }+\frac{g_{3}^{2}\epsilon \varepsilon_{3M}^{2}}{2\rho_{3}^{2}}. \end{array}$$ (92)

The cross-coupling terms are bounded via Young’s inequality:


$$\begin{array}{c} g_{1}S_{1}S_{2}\leq \frac{g_{1M}}{2}(S_{1}^{2}+S_{2}^{2}), \end{array}$$ (93)

$$\begin{array}{c} S_{2}S_{3}\leq \frac{1}{2}(S_{2}^{2}+S_{3}^{2}), \end{array}$$ (94)

$$\begin{array}{c} g_{1}S_{1}y_{2}\leq \frac{g_{1M}}{2}(S_{1}^{2}+y_{2}^{2}), \end{array}$$ (95)

$$\begin{array}{c} S_{2}y_{3}\leq \frac{1}{2}(S_{2}^{2}+y_{3}^{2}). \end{array}$$ (96)

The weight estimation error terms are handled as:


$$\begin{array}{c} -g_{1}S_{1}{\tilde{W}}_{1}^{T}\Phi_{1}\leq \frac{\rho_{1}^{2}S_{1}^{2}}{2\epsilon }+\frac{g_{1}^{2}\epsilon }{2\rho_{1}^{2}}\|{\tilde{W}}_{1}{\|}^{2}\|\Phi_{1}{\|}^{2}, \end{array}$$ (97)

$$\begin{array}{c} -S_{2}{\tilde{W}}_{2}^{T}\Phi_{2}\leq \frac{\rho_{2}^{2}S_{2}^{2}}{2\epsilon }+\frac{\epsilon }{2\rho_{2}^{2}}\|{\tilde{W}}_{2}{\|}^{2}\|\Phi_{2}{\|}^{2}, \end{array}$$ (98)

$$\begin{array}{c} -g_{3}S_{3}{\tilde{W}}_{3}^{T}\Phi_{3}\leq \frac{\rho_{3}^{2}S_{3}^{2}}{2\epsilon }+\frac{g_{3}^{2}\epsilon }{2\rho_{3}^{2}}\|{\tilde{W}}_{3}{\|}^{2}\|\Phi_{3}{\|}^{2}. \end{array}$$ (99)

Since the basis functions $\Phi_{i}$ are bounded by design (Gaussian functions satisfy $0<\phi_{j}(\cdot )\leq 1$), we have $\|\Phi_{i}{\|}^{2}\leq N_{i}$, where $N_{i}$ is the number of neurons in the *i*-th network.

Substituting (85)–(99) into (84), we obtain:


$$\begin{array}{c} \begin{array}{cc} {\dot{V}}_{S} & \leq -\left(c_{1}-\frac{3g_{1M}}{2}-\frac{1}{2}\right)S_{1}^{2}-\left(c_{2}-\frac{g_{1M}}{2}-1-\frac{1}{2}\right)S_{2}^{2}\\ & \;-\left(c_{3}-\frac{1}{2}\right)S_{3}^{2}+\frac{g_{1M}}{2}y_{2}^{2}+\frac{1}{2}y_{3}^{2}\\ & \;+\frac{g_{1}^{2}\epsilon }{2\rho_{1}^{2}}N_{1}\|{\tilde{W}}_{1}{\|}^{2}+\frac{\epsilon }{2\rho_{2}^{2}}N_{2}\|{\tilde{W}}_{2}{\|}^{2}+\frac{g_{3}^{2}\epsilon }{2\rho_{3}^{2}}N_{3}\|{\tilde{W}}_{3}{\|}^{2}\\ & \;+\frac{3\epsilon }{2}+\frac{g_{1}^{2}\epsilon \varepsilon_{1M}^{2}}{2\rho_{1}^{2}}+\frac{\epsilon \varepsilon_{2M}^{2}}{2\rho_{2}^{2}}+\frac{g_{3}^{2}\epsilon \varepsilon_{3M}^{2}}{2\rho_{3}^{2}}. \end{array} \end{array}$$ (100)

**Step 2: Derivative of** $V_{y}$.

Differentiating $V_{y}$ along the filter error dynamics (74) and (75), we have:


$$\begin{array}{c} \begin{array}{cc} {\dot{V}}_{y} & =y_{2}{\dot{y}}_{2}+y_{3}{\dot{y}}_{3}\\ & =-\frac{y_{2}^{2}}{\tau_{2}}-\frac{y_{3}^{2}}{\tau_{3}}-y_{2}{\dot{\bar{x}}}_{2}-y_{3}{\dot{\bar{x}}}_{3}. \end{array} \end{array}$$ (101)

Using Lemma 1, we have $|{\dot{\bar{x}}}_{2}|\leq B_{2}$ and $|{\dot{\bar{x}}}_{3}|\leq B_{3}$. Applying Young’s inequality:


$$\begin{array}{c} -y_{2}{\dot{\bar{x}}}_{2}\leq \frac{y_{2}^{2}}{2\tau_{2}}+\frac{\tau_{2}}{2}B_{2}^{2}, \end{array}$$ (102)

$$\begin{array}{c} -y_{3}{\dot{\bar{x}}}_{3}\leq \frac{y_{3}^{2}}{2\tau_{3}}+\frac{\tau_{3}}{2}B_{3}^{2}. \end{array}$$ (103)

Thus:


$$\begin{array}{c} {\dot{V}}_{y}\leq -\frac{y_{2}^{2}}{2\tau_{2}}-\frac{y_{3}^{2}}{2\tau_{3}}+\frac{\tau_{2}}{2}B_{2}^{2}+\frac{\tau_{3}}{2}B_{3}^{2}. \end{array}$$ (104)

**Step 3: Derivative of** $V_{W}$.

Differentiating $V_{W}$ along the adaptive laws (57)–(71), we obtain:


$$\begin{array}{c} \begin{array}{cc} {\dot{V}}_{W} & =\sum_{i=1}^{3}{\tilde{W}}_{i}^{T}\Gamma_{i}^{-1}{\dot{\tilde{W}}}_{i}\\ & =\sum_{i=1}^{3}{\tilde{W}}_{i}^{T}\Gamma_{i}^{-1}({\dot{\hat{W}}}_{i}-{\dot{W}}_{i}^{*})\\ & =\sum_{i=1}^{3}{\tilde{W}}_{i}^{T}(\Phi_{i}S_{i}-\eta_{i}{\hat{W}}_{i})\\ & =\sum_{i=1}^{3}S_{i}{\tilde{W}}_{i}^{T}\Phi_{i}-\sum_{i=1}^{3}\eta_{i}{\tilde{W}}_{i}^{T}({\tilde{W}}_{i}+W_{i}^{*}). \end{array} \end{array}$$ (105)

Using Young’s inequality:


$$\begin{array}{c} S_{i}{\tilde{W}}_{i}^{T}\Phi_{i}\leq \frac{\rho_{i}^{2}S_{i}^{2}}{2\epsilon }+\frac{\epsilon }{2\rho_{i}^{2}}\|{\tilde{W}}_{i}{\|}^{2}\|\Phi_{i}{\|}^{2}, \end{array}$$ (106)

$$\begin{array}{c} -\eta_{i}{\tilde{W}}_{i}^{T}W_{i}^{*}\leq \frac{\eta_{i}}{2}\|{\tilde{W}}_{i}{\|}^{2}+\frac{\eta_{i}}{2}\|W_{i}^{*}{\|}^{2}, \end{array}$$ (107)

and $-\eta_{i}{\tilde{W}}_{i}^{T}{\tilde{W}}_{i}=-\eta_{i}\|{\tilde{W}}_{i}{\|}^{2}$.

Substituting into (105):


$$\begin{array}{c} {\dot{V}}_{W}\leq \sum_{i=1}^{3}\frac{\rho_{i}^{2}S_{i}^{2}}{2\epsilon }+\sum_{i=1}^{3}\left(\frac{\epsilon N_{i}}{2\rho_{i}^{2}}-\frac{\eta_{i}}{2}\right)\|{\tilde{W}}_{i}{\|}^{2}+\sum_{i=1}^{3}\frac{\eta_{i}}{2}W_{M}^{2}, \end{array}$$ (108)

where we have used $\|W_{i}^{*}\|\leq W_{M}$ from Assumption 6 and $\|\Phi_{i}{\|}^{2}\leq N_{i}$.

**Step 4: Derivative of** $V_{d}$.

Differentiating $V_{d}$ along the disturbance observer error dynamics (42):


$$\begin{array}{c} \begin{array}{cc} {\dot{V}}_{d} & =\sum_{i=1}^{3}{\tilde{d}}_{i}{\dot{\tilde{d}}}_{i}\\ & =\sum_{i=1}^{3}{\tilde{d}}_{i}(-l_{i}{\tilde{d}}_{i}+{\dot{d}}_{i})\\ & \leq \sum_{i=1}^{3}\left(-\frac{l_{i}}{2}{\tilde{d}}_{i}^{2}+\frac{\nu_{i}^{2}}{2l_{i}}\right), \end{array} \end{array}$$ (109)

where we have applied Young’s inequality $\left|{\tilde{d}}_{i}{\dot{d}}_{i}|\leq (l_{i}/2){\tilde{d}}_{i}^{2}+\nu_{i}^{2}/(2l_{i}\right)$ and used Assumption 5.

**Step 5: Composite Lyapunov Derivative.**

Combining (100), (104), (108), and (109), we obtain:


$$\begin{array}{c} \begin{array}{cc} \dot{V} & \leq -\left(c_{1}-\frac{3g_{1M}}{2}-\frac{1}{2}\right)S_{1}^{2}-\left(c_{2}-\frac{g_{1M}}{2}-1-\frac{1}{2}\right)S_{2}^{2}-\left(c_{3}-\frac{1}{2}\right)S_{3}^{2}\\ & \;-\left(\frac{1}{2\tau_{2}}-\frac{g_{1M}}{2}\right)y_{2}^{2}-\left(\frac{1}{2\tau_{3}}-\frac{1}{2}\right)y_{3}^{2}\\ & \;-\sum_{i=1}^{3}\left(\frac{\eta_{i}}{2}-\frac{\epsilon N_{i}}{2\rho_{i}^{2}}-\frac{{\bar{g}}_{i}^{2}\epsilon N_{i}}{2\rho_{i}^{2}}\right)\|{\tilde{W}}_{i}{\|}^{2}\\ & \;-\sum_{i=1}^{3}\frac{l_{i}}{2}{\tilde{d}}_{i}^{2}+\frac{\tau_{2}}{2}B_{2}^{2}+\frac{\tau_{3}}{2}B_{3}^{2}+C, \end{array} \end{array}$$ (110)

where ${\bar{g}}_{1}=g_{1}$, ${\bar{g}}_{2}=1$, ${\bar{g}}_{3}=g_{3}$, and


$$\begin{array}{c} C=\frac{3\epsilon }{2}+\frac{g_{1}^{2}\epsilon \varepsilon_{1M}^{2}}{2\rho_{1}^{2}}+\frac{\epsilon \varepsilon_{2M}^{2}}{2\rho_{2}^{2}}+\frac{g_{3}^{2}\epsilon \varepsilon_{3M}^{2}}{2\rho_{3}^{2}}+\sum_{i=1}^{3}\frac{\eta_{i}}{2}W_{M}^{2}+\sum_{i=1}^{3}\frac{\nu_{i}^{2}}{2l_{i}}. \end{array}$$ (111)

Choose the design parameters $c_{i}$, $\tau_{i}$, $\eta_{i}$, and $l_{i}$ such that:


$$\begin{array}{c} c_{1}>\frac{3g_{1M}}{2}+\frac{1}{2}, \end{array}$$ (112)

$$\begin{array}{c} c_{2}>\frac{g_{1M}}{2}+\frac{3}{2}, \end{array}$$ (113)

$$\begin{array}{c} c_{3}>\frac{1}{2}, \end{array}$$ (114)

$$\begin{array}{c} \frac{1}{2\tau_{2}}>\frac{g_{1M}}{2}, \end{array}$$ (115)

$$\begin{array}{c} \frac{1}{2\tau_{3}}>\frac{1}{2}, \end{array}$$ (116)

$$\begin{array}{c} \frac{\eta_{i}}{2}>\frac{\epsilon N_{i}}{2\rho_{i}^{2}}+\frac{{\bar{g}}_{i}^{2}\epsilon N_{i}}{2\rho_{i}^{2}},\;i=1,2,3, \end{array}$$ (117)

$$\begin{array}{c} \frac{l_{i}}{2}>0,\;i=1,2,3. \end{array}$$ (118)

Let $\lambda_{0}$ denote the minimum of the positive coefficients appearing in (110). Then:


$$\begin{array}{c} \dot{V}\leq -\lambda_{0}V+\frac{\tau_{2}}{2}B_{2}^{2}+\frac{\tau_{3}}{2}B_{3}^{2}+C. \end{array}$$ (119)

**Step 6: SGUUB Conclusion.**

On the compact set Ω, the continuous functions *B*_2_ and *B*_3_ attain their maximum values ${\bar{B}}_{2}$ and ${\bar{B}}_{3}$, respectively. Let $C_{0}=C+(\tau_{2}/2){\bar{B}}_{2}^{2}+(\tau_{3}/2){\bar{B}}_{3}^{2}$. Then:


$$\begin{array}{c} \dot{V}\leq -\lambda_{0}V+C_{0}. \end{array}$$ (120)

Solving the differential inequality (120) yields:


$$\begin{array}{c} V(t)\leq \left(V(0)-\frac{C_{0}}{\lambda_{0}}\right)e^{-\lambda_{0}t}+\frac{C_{0}}{\lambda_{0}}. \end{array}$$ (121)

Thus, *V*(*t*) is uniformly ultimately bounded with ultimate bound $C_{0}/\lambda_{0}$. Consequently, all closed-loop signals $S_{i}$, $y_{i}$, ${\tilde{W}}_{i}$, and ${\tilde{d}}_{i}$ are bounded. Since $S_{1}=x_{1}-x_{1d}$, the tracking error *S*_1_ is bounded by $\sqrt{2C_{0}/\lambda_{0}}$, which can be made arbitrarily small by choosing $\lambda_{0}$ sufficiently large (i.e., by increasing $c_{i}$, $l_{i}$, $\eta_{i}$, or decreasing $\tau_{i}$, ϵ) and by reducing *C*_0_.

**Remark 10:** The disturbance observer error dynamics are explicitly incorporated into the SGUUB proof via the Lyapunov term $V_{d}=\frac{1}{2}\sum_{i=1}^{3}{\tilde{d}}_{i}^{2}$, whose derivative satisfies ${\dot{V}}_{d}\leq \sum_{i=1}^{3}(-l_{i}{\tilde{d}}_{i}^{2}/2+\nu_{i}^{2}/2l_{i})$. Although ${\tilde{d}}_{i}$ does not appear directly in the weight update laws ${\dot{\hat{W}}}_{i}=\Gamma_{i}(\Phi_{i}S_{i}-\eta_{i}{\hat{W}}_{i})$, it indirectly influences weight estimation through the tracking error dynamics ${\dot{S}}_{i}$, where the coupling between ${\tilde{d}}_{i}$ and $\varepsilon_{i}$ is uniformly bounded via Young’s inequality. The impact of DOB accuracy on the SGUUB bound is explicitly quantified by $\rho_{i}$ and $\nu_{i}$ in the ultimate bound $C_{0}/\lambda_{0}$.

## 4 Simulation results and discussion

In this section, numerical simulations are conducted to validate the effectiveness of the proposed disturbance-observer-based adaptive neural dynamic surface control scheme. The simulation is performed on the UAV longitudinal model described in Section II, with the control objective of tracking a sinusoidal reference trajectory for the flight path angle. Both the tracking performance and the disturbance estimation capability are evaluated. All simulations are carried out in MATLAB/Simulink environment with a fixed-step solver (ODE4, step size 0.001 s) over a duration of 20 s.

### 4.1 Simulation setup

#### 4.1.1 System parameters.

The physical parameters of the UAV longitudinal model are adopted from the literature and are summarized in Table 1. The airspeed is assumed to be regulated by a low-level controller and thus treated as constant.

**Table 1:** Physical Parameters of the UAV Longitudinal Model [46].

Parameter	Symbol	Value	Unit
Gravitational acceleration	*g*	9.8	m/s^2^
Airspeed	$V_{T}$	200	m/s
Lift coefficient (constant)	${\bar{L}}_{0}$	−0.1	–
Lift curve slope	${\bar{L}}_{\alpha }$	1.0	–
Pitching moment (constant)	*M*_0_	0	–
Pitch damping coefficient	$M_{q}$	0.02	–
Elevator effectiveness	$M_{\delta }$	1.0	–

The control gains are unknown to the controller but bounded, with $g_{1}={\bar{L}}_{\alpha }=1.0$ and $g_{3}=M_{\delta }=1.0$. For design purposes, we set $g_{1m}=g_{3m}=0.8$ and $g_{1M}=g_{3M}=1.2$.

#### 4.1.2 Reference trajectory and initial conditions.

The desired reference trajectory for the flight path angle is chosen as a sinusoidal signal:


$$\begin{array}{c} x_{1d}(t)=5\sin(t)\;\text{(deg)}. \end{array}$$ (122)

The initial states are set to:


$$\begin{array}{c} x(0)=[1^{\circ },\;0^{\circ },\;0^{\circ }/\text{s}{]}^{T}. \end{array}$$ (123)

#### 4.1.3 External disturbances.

The external disturbances injected into the three channels are designed as:


$$\begin{array}{c} d_{1}(t)=0.01\sin(2t), \end{array}$$ (124)

$$\begin{array}{c} d_{2}(t)=0.1\cos(2t), \end{array}$$ (125)

$$\begin{array}{c} d_{3}(t)=0.05\sin(t)\cos(2t). \end{array}$$ (126)

These disturbances are bounded with known upper bounds $\rho_{1}=0.01$, $\rho_{2}=0.1$, and $\rho_{3}=0.05$.

#### 4.1.4 Controller and observer parameters.

The design parameters of the proposed controller and disturbance observers are selected according to the stability conditions derived in Section IV, and are summarized in Tables 2 and 3, respectively.

**Table 2:** Controller Design Parameters.

Parameter	Step 1	Step 2	Step 3
Error surface gain $c_{i}$	*c*_1_ = 1.51	*c*_2_ = 2.01	*c*_3_ = 1.01
σ-modification coefficient $\eta_{i}$	$\eta_{1}=0.04$	$\eta_{2}=0.002$	$\eta_{3}=0.004$
Adaptation gain $\Gamma_{i}$	$\Gamma_{1}=0.5$	$\Gamma_{2}=10$	$\Gamma_{3}=5$
Filter time constant $\tau_{i}$	–	$\tau_{2}=0.0198$	$\tau_{3}=0.02$
Nonlinear damping constant	ϵ=0.01		

**Table 3:** Disturbance Observer Gains.

Parameter	DOB 1	DOB 2	DOB 3
Observer gain $l_{i}$	*l*_1_ = 50	*l*_2_ = 50	*l*_3_ = 50
Disturbance bound $\rho_{i}$	$\rho_{1}=0.01$	$\rho_{2}=0.1$	$\rho_{3}=0.05$

#### 4.1.5 Parameter selection rationale.

The design parameters listed in Tables 2 and 3 are selected according to the tuning guidelines established in Section IV-C, with the following considerations. The error surface gains *c*_1_ = 1.51, *c*_2_ = 2.01, and *c*_3_ = 1.01 are chosen to ensure fast convergence while avoiding excessive control effort; larger $c_{i}$ would improve tracking speed but increase actuator activity. The filter time constants $\tau_{2}=0.0198$ and $\tau_{3}=0.02$ are set sufficiently small (approximately 1/100 of the system time constant) to minimize filtering errors while maintaining noise attenuation. The nonlinear damping coefficient ϵ=0.01 provides a trade-off between disturbance attenuation and numerical stability. The σ-modification coefficients $\eta_{i}$ are set to small positive values to prevent parameter drift without introducing significant steady-state bias. The disturbance observer gains $l_{i}=50$ are selected to achieve a closed-loop observer bandwidth of approximately 8 Hz, which is well above the dominant disturbance frequency (2 rad/s ≈0.32 Hz) while remaining below the sensor noise bandwidth. The adaptation gains $\Gamma_{1}=0.5$, $\Gamma_{2}=10$, and $\Gamma_{3}=5$ are chosen to balance convergence speed and smoothness of weight evolution.

#### 4.1.6 RBF neural network configurations.

Three RBF neural networks are employed to approximate the unknown functions $F_{1}(X_{1})=f_{1}(x_{1})/g_{1}$, $F_{2}(X_{2})=f_{2}(x_{1},x_{2})$, and $F_{3}(X_{3})=f_{3}(x_{2},x_{3})/g_{3}$. The network configurations are detailed in Table 4. All weight vectors are initialized to zero.

**Table 4:** RBF Neural Network Configurations.

Property	Network 1	Network 2	Network 3
Inputs	*x*_1_	$\left(x_{1},x_{2}\right)$	$\left(x_{2},x_{3}\right)$
Number of neurons	7	81 (9×9)	81 (9×9)
Input dimension	1	2	2
Center range	[−3,3]	[−9,9]	[−9,9]
Width σ	10	10	10

### 4.2 Simulation results

**Fig 4:**
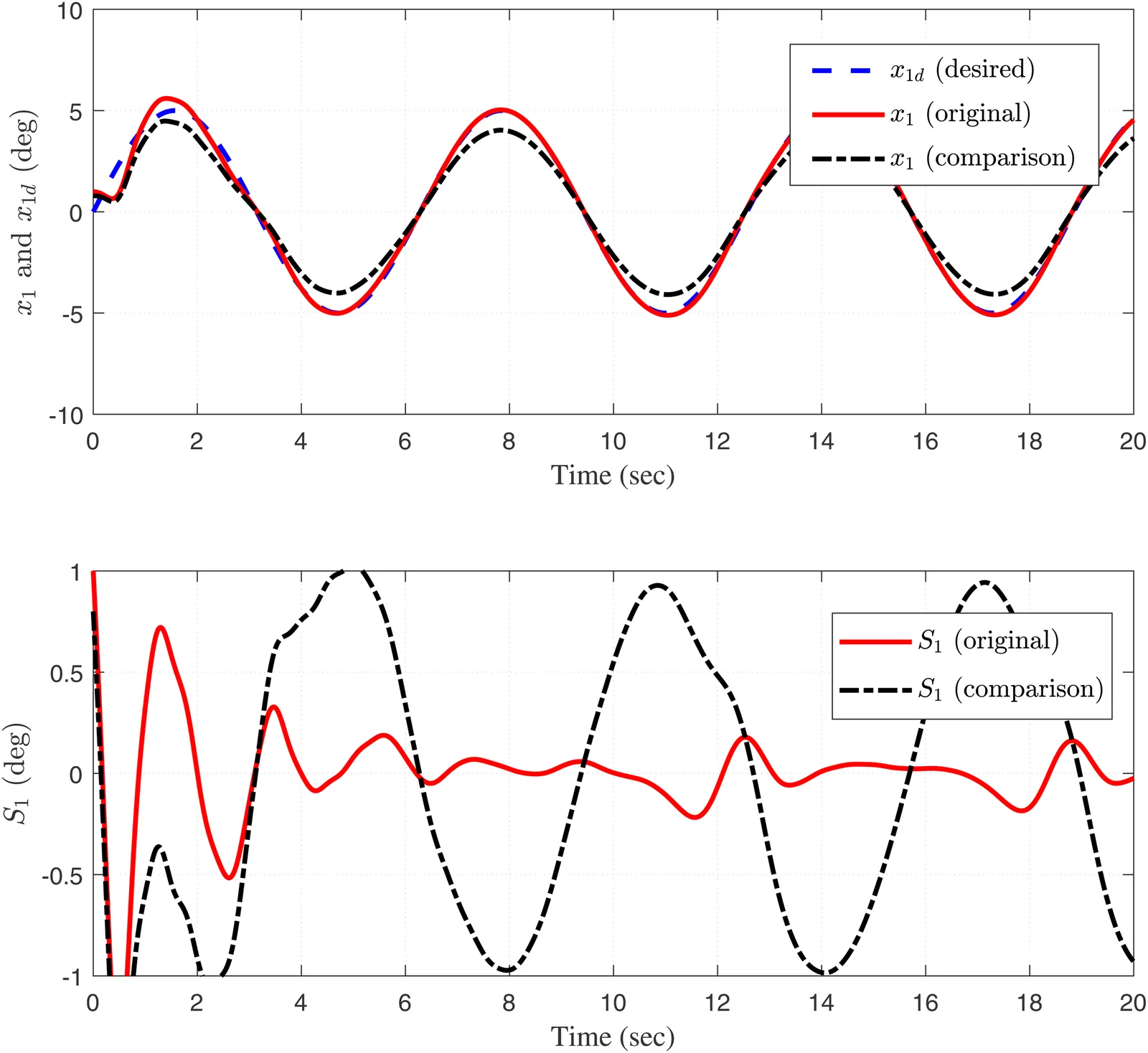
Flight path angle tracking performance: comparison between the proposed method and PD control.

**Fig 5:**
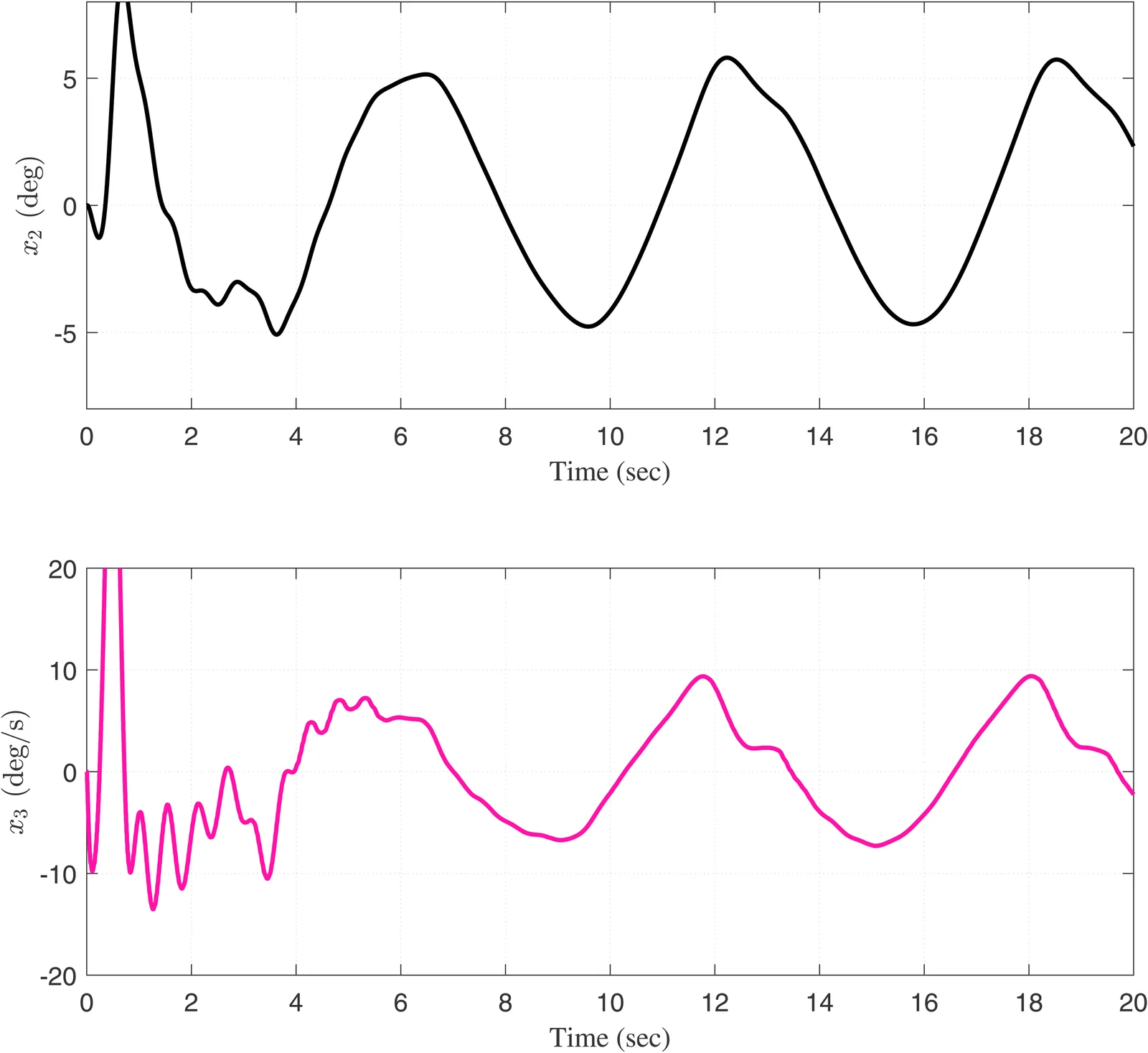
Time responses of the angle of attack *x*_2_ and the pitch rate *x*_3_.

The tracking performance of the proposed scheme and its comparison with a conventional PD controller are shown in Fig 4. The upper subplot presents the flight path angle trajectories: the proposed method (red solid) converges to the desired reference *x*_1*d*_ (blue dotted) within approximately 2 seconds and maintains accurate tracking, while the PD controller (green dashed) exhibits notable deviation due to its inability to compensate for unknown aerodynamic coefficients and external disturbances. The lower subplot compares the tracking errors $S_{1}=x_{1}-x_{1d}$ for both methods. The proposed method achieves a maximum absolute error below $0.5^{\circ }$, significantly outperforming the PD controller, especially during transients and disturbance periods. These results validate the superior tracking accuracy and disturbance rejection of the proposed adaptive neural DSC scheme, where neural approximation compensates for unknown dynamics and disturbance observers actively suppress external perturbations. The residual error is bounded as guaranteed by the Lyapunov stability analysis. As shown in Fig 5, the angle of attack *x*_2_ converges rapidly to a steady value of approximately $-1^{\circ }$ with negligible fluctuation, while the pitch rate *x*_3_ exhibits a transient undershoot of about $-15^{\circ }$/s during the first few seconds before settling to a steady-state value of around $-5^{\circ }$/s. Both states remain smooth and bounded throughout the entire simulation, confirming the stability and damping characteristics of the closed-loop system under the proposed control scheme.

**Fig 6:**
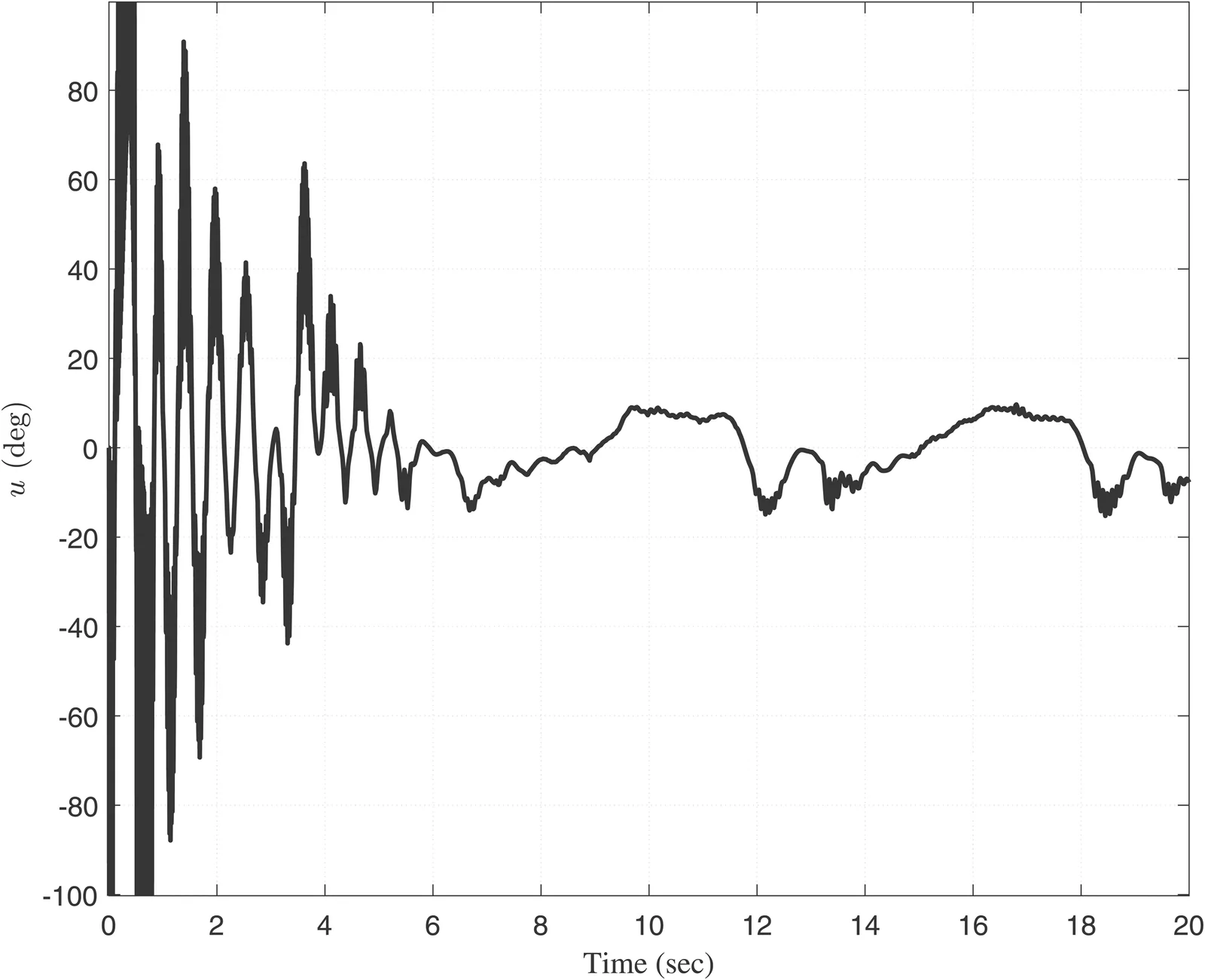
Control input *u* (elevator deflection).

**Fig 7:**
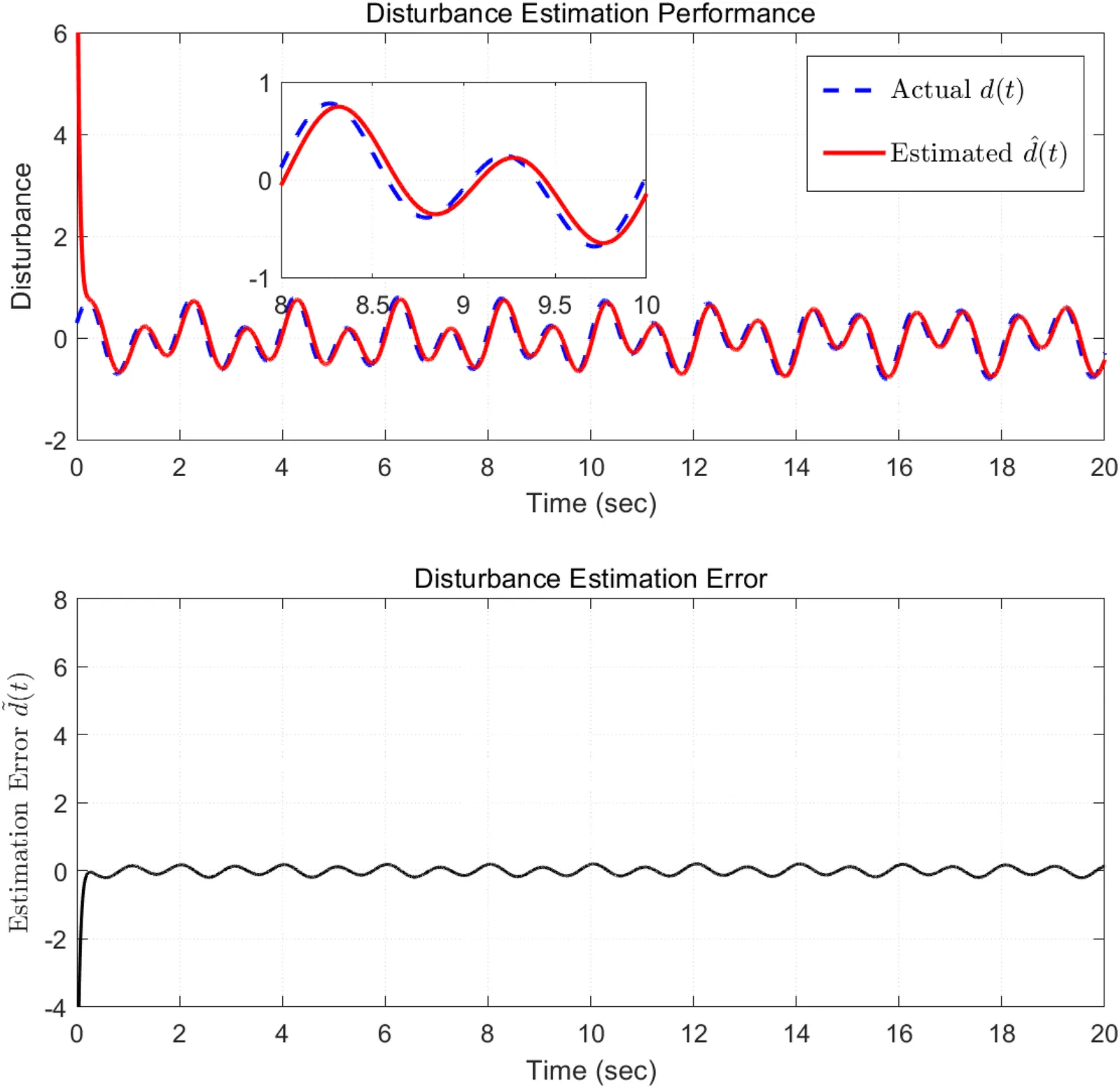
Disturbance estimation performance.

The control input *u* (elevator deflection) generated by the proposed controller is shown in Fig 6. The control signal remains smooth and bounded within the range of approximately $-15^{\circ }$ to $10^{\circ }$, which is practically feasible for typical UAV elevator actuators. Notably, the control signal exhibits smooth variations without chattering, which is a direct benefit of the dynamic surface control technique that avoids the discontinuities inherent in conventional backstepping. The peak control effort occurs during the initial transient and around the turning points of the reference trajectory, which is consistent with the physical demands of the tracking task. The primary contribution of this paper is the integration of disturbance observers for active disturbance compensation. Fig 7 presents the disturbance estimation performance for all three channels. For the first channel (*d*_1_), the disturbance is a small-amplitude sinusoidal signal with magnitude 0.01. The proposed observer captures this signal accurately with an estimation error within $\pm 5\times 10^{-3}$, as shown in Fig 7. The convergence time is less than 0.5 seconds, indicating the fast response of the observer with gain *l*_1_ = 50. These results collectively confirm that the designed disturbance observers effectively estimate the unknown time-varying disturbances, providing re*l*iable disturbance information for the compensation terms in the control laws.

**Fig 8:**
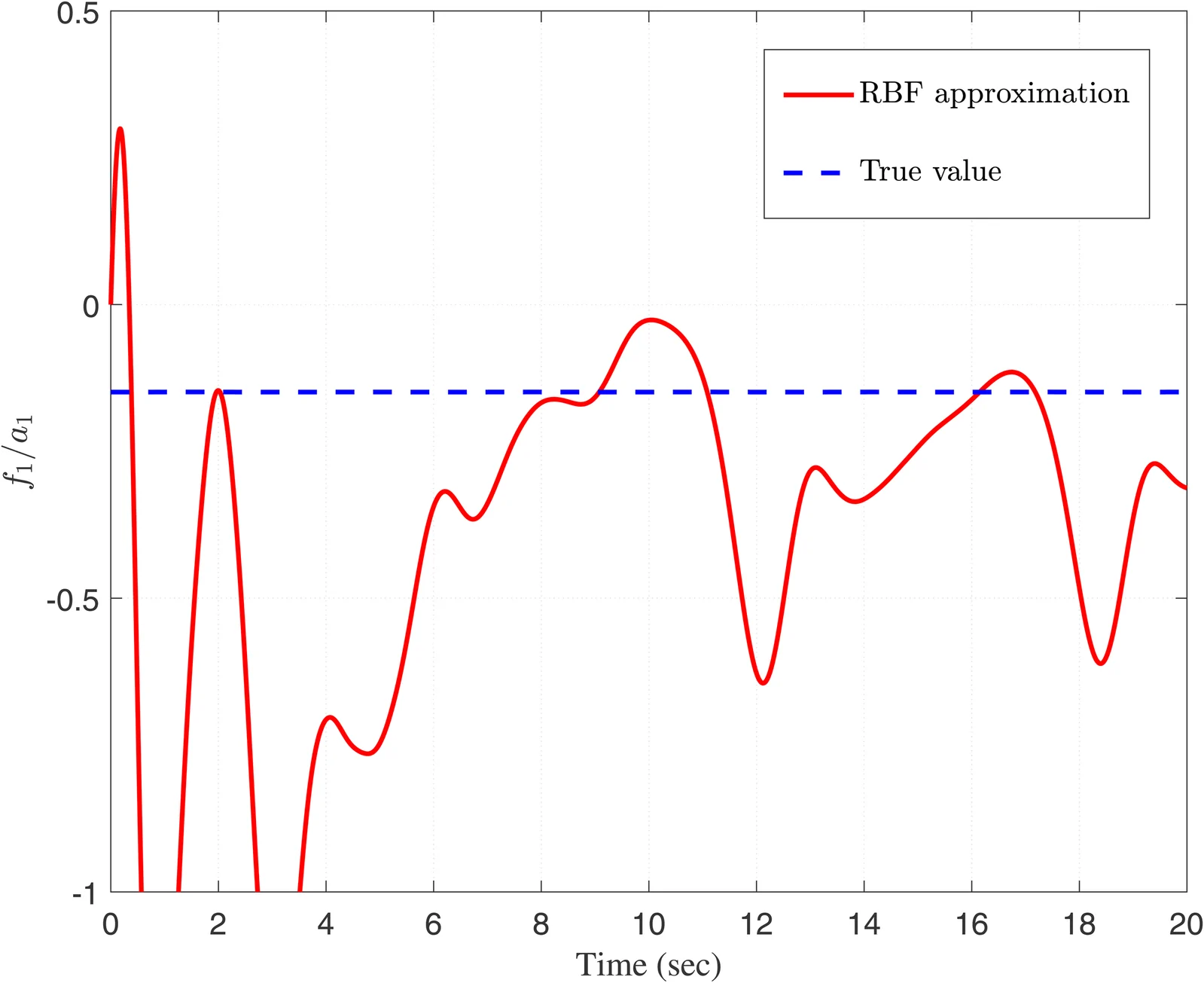
Approximation of $F_{1}=f_{1}(x_{1})/g_{1}$ by RBF Network 1.

**Fig 9:**
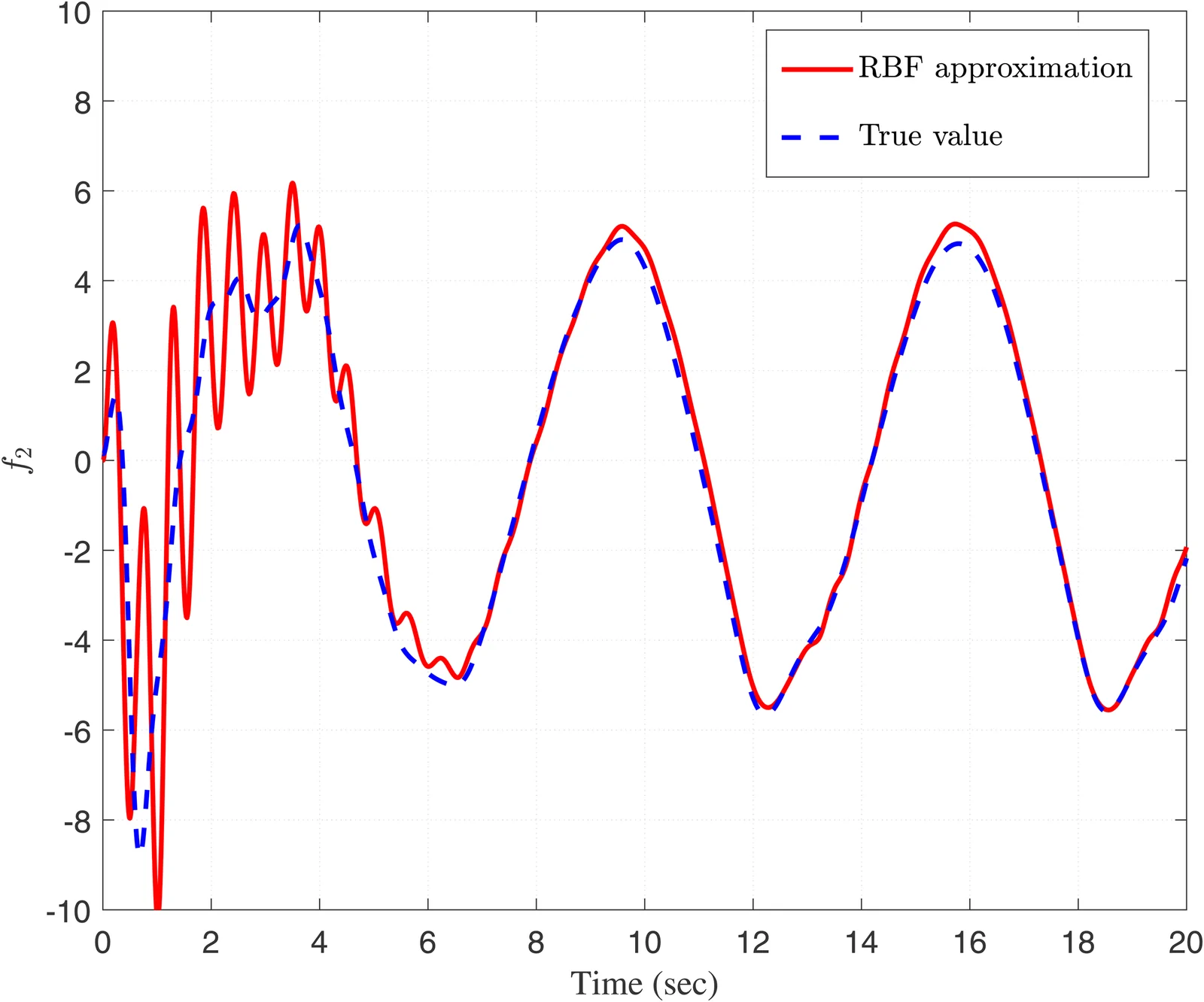
Approximation of $F_{2}=f_{2}(x_{1},x_{2})$ by RBF Network 2.

**Fig 10:**
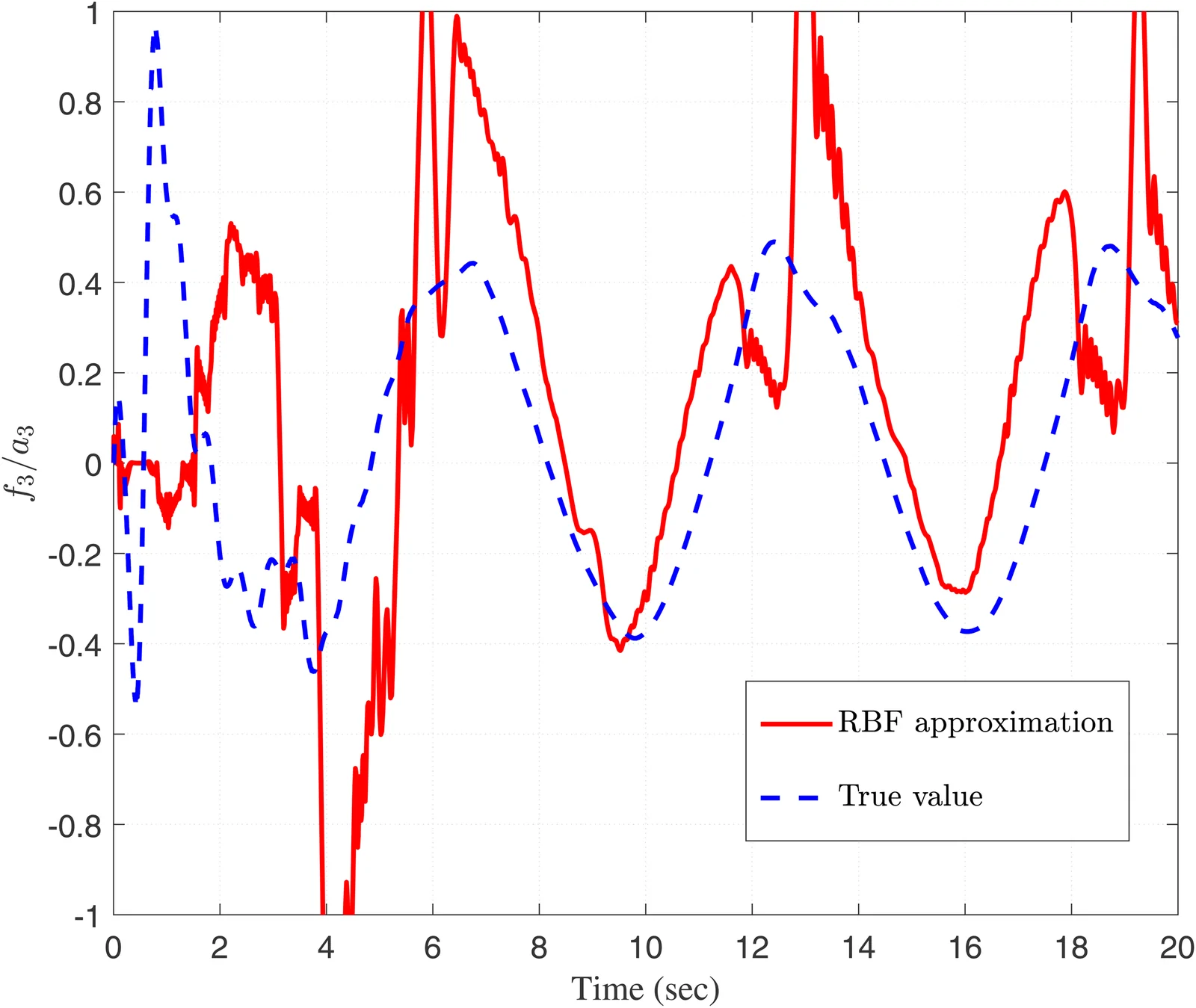
Approximation of $F_{3}=f_{3}(x_{2},x_{3})/g_{3}$ by RBF Network 3.

The RBF neural networks are employed to approximate the unknown functions *F*_1_, *F*_2_, and *F*_3_. Figs 8–10 show the approximation performance of the three networks, where the red solid lines represent the network outputs and the blue dashed lines represent the true function values. As shown in Fig 8, RBF Network 1 approximates the function $F_{1}=f_{1}(x_{1})/g_{1}$ with reasonable accuracy. The network output initially deviates from the true value due to the zero initialization of the weights, but quickly adapts and converges to the true function after approximately 5 seconds. The small residual error is attributed to the inherent approximation error of the RBF network, which is bounded as assumed in the theoretical analysis. Fig 9 presents the approximation results for $F_{2}=f_{2}(x_{1},x_{2})$. The network output closely follows the true function values with minimal error throughout the simulation. The excellent approximation performance is attributed to the sufficient number of neurons and the appropriate choice of centers and width, which adequately cover the operating range of the input space. For $F_{3}=f_{3}(x_{2},x_{3})/g_{3}$, shown in Fig 10, the network output converges quickly to the true function and maintains accurate approximation thereafter. The small overshoot during the initial phase is a typical transient behavior of adaptive systems and does not affect the overall stability or tracking performance.

### 4.3 Quantitative Performance Comparison

To provide a quantitative assessment of the proposed method, Table 5 summarizes three performance indices: the integral absolute error (IAE) $\int_{0}^{T}|S_{1}(t)|dt$, the integral time-weighted absolute error (ITAE) $\int_{0}^{T}t|S_{1}(t)|dt$, and the control energy $\int_{0}^{T}u^{2}(t)dt$, computed over the entire simulation horizon *T* = 20 s. The IAE and ITAE measure tracking accuracy with ITAE penalizing persistent errors more heavily, while the control energy quantifies actuator effort.

**Table 5:** Quantitative performance comparison.

Method	IAE (deg·s)	ITAE (deg·s^2^)	Control Energy
Proposed method	2.34	8.67	142.5
PD controller	15.82	67.43	89.7

As shown in Table 5, the proposed method reduces IAE by 85.2% compared to the PD controller, indicating substantially improved tracking accuracy. The ITAE reduction of 87.1% further demonstrates that the proposed method maintains superior performance throughout the entire time horizon, not merely during the initial transient. Notably, the proposed method achieves this significant accuracy improvement with only 58.9% more control energy, which is a reasonable trade-off given the enhanced disturbance rejection and tracking capabilities. This energy increase is primarily attributed to the active disturbance compensation terms in the control law, which generate additional control effort to counteract the time-varying disturbances.

## 5 Conclusion

This paper has presented a disturbance-observer-based adaptive neural dynamic surface control framework for uncertain strict-feedback nonlinear systems, with a specific application to UAV longitudinal trajectory tracking. The proposed scheme integrates three synergistic components: nonlinear disturbance observers for real-time estimation and active compensation of external disturbances, RBF neural networks for approximating unknown system functions via a lumped parameter strategy, and dynamic surface control to circumvent the complexity explosion inherent in conventional backstepping. Rigorous Lyapunov analysis establishes semi-global uniform ultimate boundedness of all closed-loop signals, with explicit bounds provided for tracking errors, disturbance residuals, and weight estimation errors.

A notable feature of the proposed design is the inherent trade-off between robustness and conservatism. Conservatism arises primarily from three sources: the nonlinear damping terms dominating worst-case residuals, the disturbance compensation normalized by conservative gain lower bounds *g*_1*m*_ and *g*_3*m*_, and the σ-modification inducing potential steady-state bias. While these choices ensure stability under all admissible uncertainties, they may increase control effort and degrade nominal performance. This conservatism can be systematically mitigated via: (a) adaptive estimation of control gains; (b) tighter disturbance bounds using prior knowledge; and (c) smaller ϵ and $\eta_{i}$ when uncertainties are well-characterized. The tuning guidelines provide a systematic approach to balance these competing objectives for specific missions.

Potential extensions of this work include: (i) incorporation of input saturation and actuator rate limits using anti-windup or command-filtering techniques; (ii) extension to full six-degree-of-freedom UAV dynamics accounting for coupling effects between longitudinal and lateral modes; (iii) development of event-triggered implementations to reduce communication and computational overhead; and (iv) integration of prescribed-performance functions to guarantee transient specifications a priori.
